# Genes Associated with the Flax Plant Type (Oil or Fiber) Identified Based on Genome and Transcriptome Sequencing Data

**DOI:** 10.3390/plants10122616

**Published:** 2021-11-28

**Authors:** Liubov V. Povkhova, Nataliya V. Melnikova, Tatiana A. Rozhmina, Roman O. Novakovskiy, Elena N. Pushkova, Ekaterina M. Dvorianinova, Alexander A. Zhuchenko, Anastasia M. Kamionskaya, George S. Krasnov, Alexey A. Dmitriev

**Affiliations:** 1 Engelhardt Institute of Molecular Biology, Russian Academy of Sciences, 119991 Moscow, Russia; povhova.lv@phystech.edu (L.V.P.); mnv-4529264@yandex.ru (N.V.M.); 0legovich46@mail.ru (R.O.N.); pushkova18@gmail.com (E.N.P.); dvorianinova.em@phystech.edu (E.M.D.); gskrasnov@mail.ru (G.S.K.); 2 Moscow Institute of Physics and Technology, 141701 Moscow, Russia; 3 Federal Research Center for Bast Fiber Crops, 172002 Torzhok, Russia; tatyana_rozhmina@mail.ru (T.A.R.); ecovilar@mail.ru (A.A.Z.); 4 All-Russian Horticultural Institute for Breeding, Agrotechnology and Nursery, 115598 Moscow, Russia; 5 Institute of Bioengineering, Research Center of Biotechnology of the Russian Academy of Sciences, 119071 Moscow, Russia; rifampicin@yandex.ru

**Keywords:** flax, *Linum usitatissimum* L., fiber, seeds, genome and transcriptome sequencing, polymorphisms, gene expression

## Abstract

As a result of the breeding process, there are two main types of flax (*Linum usitatissimum* L.) plants. Linseed is used for obtaining seeds, while fiber flax is used for fiber production. We aimed to identify the genes associated with the flax plant type, which could be important for the formation of agronomically valuable traits. A search for polymorphisms was performed in genes involved in the biosynthesis of cell wall components, lignans, fatty acids, and ion transport based on genome sequencing data for 191 flax varieties. For 143 of the 424 studied genes (*4CL*, *C3′H*, *C4H*, *CAD*, *CCR*, *CCoAOMT*, *COMT*, *F5H*, *HCT*, *PAL*, *CTL*, *BGAL*, *ABC*, *HMA*, *DIR*, *PLR*, *UGT*, *TUB*, *CESA*, *RGL*, *FAD*, *SAD*, and *ACT* families), one or more polymorphisms had a strong correlation with the flax type. Based on the transcriptome sequencing data, we evaluated the expression levels for each flax type-associated gene in a wide range of tissues and suggested genes that are important for the formation of linseed or fiber flax traits. Such genes were probably subjected to the selection press and can determine not only the traits of seeds and stems but also the characteristics of the root system or resistance to stresses at a particular stage of development, which indirectly affects the ability of flax plants to produce seeds or fiber.

## 1. Introduction

Flax (*Linum usitatissimum* L.) is traditionally grown for obtaining fiber from stems and oil from seeds [1,2]. The differences in the use of flax products resulted in the appearance of two main varieties: fiber flax and oil flax (linseed). Compared to linseed, fiber flax is taller, has branches only in the upper part of the plant, and produces fewer seeds with lower weight [3]. In addition, fiber flax varieties predominantly have a higher fiber content than oil flax [4]. For linseed, agronomically important traits are associated with seed characteristics, such as size, yield, and biochemical composition, while for fiber flax, fiber properties that attract attention include yield, tensile strength, density, flexibility, and biochemical composition (including the cellulose and lignin contents). Linseed is rich in unsaturated fatty acids (primarily omega-3) and lignans, which are beneficial for health and reduce the risk of cancer and cardiac diseases and is, therefore, used in pharmaceutical and food products as well as animal feed; flax oil is also a component of paints and varnishes [1,5,6,7,8,9,10,11,12,13,14]. Flax bast fiber is rich in cellulose and low in lignin, which makes fiber flax a good source for the production of high-quality textiles, medicine, and promising composite materials for the automobile, aerospace, and packaging industries [12,15,16,17,18,19,20,21,22,23].

In recent decades, a significant number of studies have aimed to understand the molecular–genetic determination of flax traits. The flax genome of cultivar CDC Bethune was sequenced and assembled to chromosomes (about 316 Mb for 15 chromosomes) [24,25]. Chromosome-level and scaffold-level de novo genome assemblies were also obtained for several flax varieties [26,27,28]. The identification of genes/genetic markers associated with agronomically important characteristics was performed using SSR (simple sequence repeats) markers [29], reduced-representation genome sequencing [30,31,32,33,34,35], whole-genome resequencing [36], combined genome-wide association analysis and transcriptome sequencing [37], and a functional approach based on the analysis of gene expression and quality parameters [38]. Transcriptomic studies of flax, including the analysis of gene and microRNA expression in particular tissues, were also carried out for the identification of genes and microRNAs that are important for flax development [37,38,39,40,41,42,43,44,45] and responses to stresses [46,47,48,49,50,51,52,53,54,55]. These studies have made a substantial contribution to our knowledge of the genetic basis of flax traits and have also created significant datasets that can be used in further research.

In addition, studies have identified and characterized particular gene families that likely participate in flax plant processes, including those involved in the biosynthesis of lignin (*4CLs* encode 4-coumarate:CoA ligases; *C3′Hs* encode p-coumarate 3-hydroxylases; *C4Hs* encode cinammate 4-hydroxylases; *CADs* encode cinnamyl alcohol dehydrogenases; *CCRs* encode cinnamoyl CoA reductases; *CCoAOMTs* encode affeoyl CoA 3-O-methyltransferases; *COMTs* encode caffeate/5-hydroxyferulate O-methyl-transferases; *F5Hs* encode 5-hydroxylases; *HCTs* encode hydroxycinnamoyl-CoA:shikimate hydroxycinnamoyl transferases; and *PALs* encode phenylalanine ammonia-lyases) [53,56] and other cell wall components (*CTLs* encode chitinase-like proteins; *BGALs* encode β-galactosidase-like proteins; *TUBs* encode tubulins; *CESAs* encode cellulose synthases; *RGLs* encode rhamnogalacturonan lyases; and *ACTs* encode actins) [42,57,58,59,60,61,62,63,64], the biosynthesis of lignans (*DIRs* encode dirigent proteins; *PLRs* encode pinoresinol–lariciresinol reductases; and *UGTs* encode UDP-glycosyltransferases) [65,66,67,68], biosynthesis of fatty acids (*FADs* encode fatty acid desaturases and *SADs* encode stearoyl-ACP desaturases) [69,70,71,72], and the transport of ions, lipids, and carbohydrates (*ABCs* encode ATP binding cassette transporters and *HMAs* encode heavy metal-associated proteins) [73]. However, it is still not clear which genes from particular families play key roles in the determination of important flax plant characteristics, which polymorphisms have the greatest impact on agronomic traits, and how gene expression affects the manifestation of a trait. A complex approach should be used to solve these issues, and novel and previously obtained data should be included in the analysis. The present study aimed to identify genes that are likely essential for the formation of a particular type of flax plant (fiber flax or linseed) based on genome and transcriptome sequencing data.

## 2. Results

### 2.1. Gene Polymorphisms

In the present work, we focused our attention on genes that are important for cell wall formation, because these genes are crucial for the determination of flax stem traits; genes involved in the biosynthesis of lignans and fatty acids, the content of which is an important characteristic of seeds; and ABC transporter and heavy metal–associated genes, which participate in numerous processes in flax plants. We performed a search for polymorphisms among 424 genes (66 lignin biosynthesis genes (*4CL*, *C3′H*, *C4H*, *CAD*, *CCR*, *CCoAOMT*, *COMT*, *F5H*, *HCT*, and *PAL*), 35 *CTL* genes, 40 *BGAL* genes, 206 *ABC* and *HMA* genes, 9 lignan biosynthesis genes (*DIR*, *PLR*, and *UGT*), 21 *TUB* genes, 16 *CESA* genes, 10 *RGL* genes, 6 fatty acid biosynthesis genes (*FAD* and *SAD*), and 15 *ACT* genes) based on genome sequencing data for 191 flax varieties (79 fiber flax and 112 linseed varieties) from the NCBI database (PRJNA590636 and PRJNA478805). The results are presented in Supplementary Table S1.

**Figure 1:**
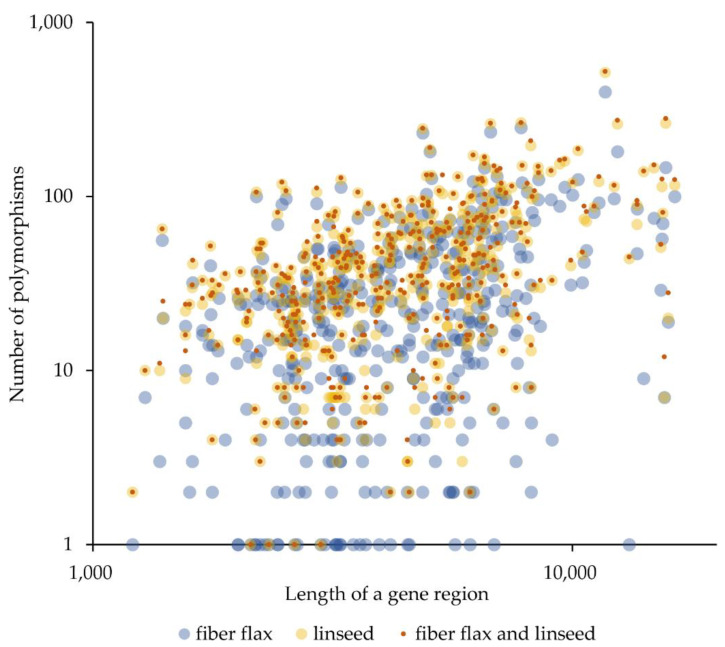
Data on the number of polymorphisms for individual genes of the *4CL*, *C3′H*, *C4H*, *CAD*, *CCR*, *CCoAOMT*, *COMT*, *F5H*, *HCT*, *PAL*, *CTL*, *BGAL*, *ABC*, *HMA*, *DIR*, *PLR*, *UGT*, *TUB*, *CESA*, *RGL*, *FAD*, *SAD*, and *ACT* families taking into account the length of the analyzed region (gene length + 1000 bp, 500 bp upstream and 500 bp downstream).

For lignin-related genes, we identified 2703 polymorphisms; for *CTL* genes, 1053; for *BGAL* genes, 2531; for *ABC* and *HMA* genes, 12,598; for lignan-related genes, 559; for *TUB* genes, 585; for *CESA* genes, 671; for *RGL* genes, 546; for fatty acid-related genes, 304; and for *ACT* genes, 208. Data on the number of polymorphisms for individual genes taking into account the length of the analyzed region (gene length + 1000 bp, 500 bp upstream and 500 bp downstream) are presented in Figure 1 and Supplementary Table S2.

**Figure 2:**
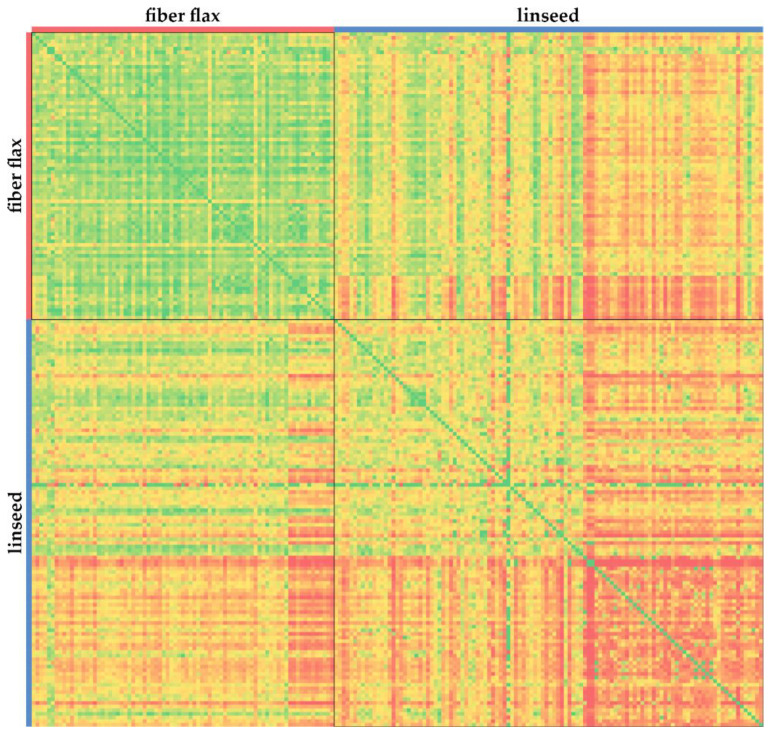
Genetic similarity between fiber flax and linseed varieties based on polymorphisms in the studied genes of the *4CL*, *C3′H*, *C4H*, *CAD*, *CCR*, *CCoAOMT*, *COMT*, *F5H*, *HCT*, *PAL*, *CTL*, *BGAL*, *ABC*, *HMA*, *DIR*, *PLR*, *UGT*, *TUB*, *CESA*, *RGL*, *FAD*, *SAD*, and *ACT* families. The green–yellow–red color scale indicates the level of genetic similarity between varieties, from the highest (green) to the lowest (red).

The most polymorphic genes were *ABCG11*, *F5H8*, *ABCH10*, *CTL9*, *HMA6*, *F5H7*, *ABCG68*, *4CL5*, *ABCG47*, and *ABCG16* (number of polymorphisms per 1 kb ranged from 37.8 to 50.6, Supplementary Table S2). As can be seen from Figure 1 and Supplementary Table S2, there were fewer polymorphisms in the analyzed fiber flax varieties than in the linseed varieties. We evaluated the genetic similarity in fiber flax and linseed groups based on polymorphisms in the studied 424 genes and confirmed that the linseed group was more polymorphic (Figure 2 and Supplementary Table S3).

**Figure 3:**
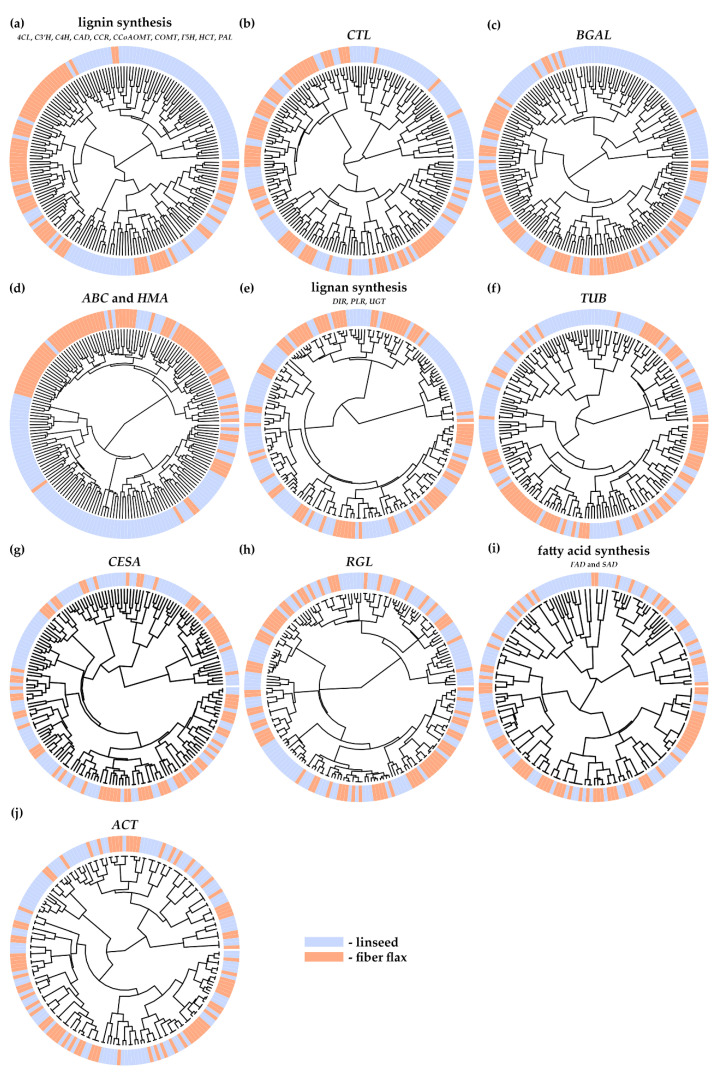
Clustering of linseed and fiber flax varieties based on polymorphisms in lignin-associated (*4CL*, *C3′H*, *C4H*, *CAD*, *CCR*, *CCoAOMT*, *COMT*, *F5H*, *HCT*, and *PAL*) (**a**), *CTL* (**b**), *BGAL* (**c**), *ABC* and *HMA* (**d**), lignan-associated (*DIR*, *PLR*, and *UGT*) (**e**), *TUB* (**f**), *CESA* (**g**), *RGL* (**h**), fatty acid–associated (*FAD* and *SAD*) (**i**), and *ACT* (**j**) gene families.

Then, clustering of flax varieties was performed based on the identified polymorphisms for the studied gene families; the results are presented in Figure 3 and Supplementary Figure S1.

For genes involved in lignin synthesis, three clusters were revealed: the first included predominantly linseed varieties; the second, predominantly fiber flax varieties; and the third, both linseed and fiber flax varieties (Figure 3a and Supplementary Figure S1a). For *CTL* genes, we also observed a cluster with the predominance of linseed, a cluster with the prevalence of fiber flax, and a mixed cluster (Figure 3b and Supplementary Figure S1b). For *BGAL* genes, sufficiently clear differentiation of flax varieties for a linseed group and a fiber flax group was revealed (Figure 3c and Supplementary Figure S1c); the same was observed for *ABC* and *HMA* genes (Figure 3d and Supplementary Figure S1d). For lignan-related genes, a cluster of predominantly linseed varieties emerged; the second cluster included mostly fiber flax varieties, while in the third cluster, differentiation according to flax type was not that clear (Figure 3e and Supplementary Figure S1e). The same was observed for *TUB* genes (Figure 3f and Supplementary Figure S1f). For *CESA* genes and *RGL* genes, association with flax type was revealed for some subclusters, but the majority of varieties formed mixed clusters (Figure 3g,h and Supplementary Figure S1g,h). For fatty acid–associated genes, a cluster of mostly linseed varieties and a cluster of mostly fiber flax ones were formed; however, each cluster included a significant number of varieties of the other type (Figure 3i and Supplementary Figure S1i). For actin genes, there was not clear enough differentiation of fiber flax and linseed varieties (Figure 3j and Supplementary Figure S1j). Thus, with some exceptions, clustering of fiber flax and linseed varieties was observed for most groups of studied genes, and the differentiation based on polymorphisms of *BGAL* genes and *ABC* plus *HMA* genes was considered the best (Figure 3).

### 2.2. Analysis of Genes Associated with Flax Type

#### 2.2.1. Genes with Flax Type–Associated Polymorphisms

For the identification of genes potentially associated with flax type, correlation analysis between variant allele frequency (VAF) values and belonging of a variety to a linseed or fiber flax group was performed. For 143 of the 424 studied genes, one or more polymorphisms had a Spearman’s correlation coefficient (r_s_) value ≥ 0.4 or ≤−0.4 with flax type (Supplementary Table S4). Data on the number of flax type-associated (FTA) polymorphisms for a particular gene are presented in Table 1. The number of polymorphisms with a high correlation coefficient significantly varied among the studied genes, ranging from 1 to 39. Several genes had a very strong correlation with flax type (r_s_ ≥ 0.6 or ≤−0.6): *PAL1*, *PAL3*, *BGAL30*, *ABCB40*, *ABCB42*, *ABCB45*, *ABCB47*, *ABCC4*, *ABCG8*, *ABCG79*, *ABCH1*, *HMA12*, *PLR1*, and *CESA1-B.* These genes could play an important role in the formation of traits specific to linseed or fiber flax and are of particular interest for further analysis.

The proportion of genes with polymorphisms with a strong correlation (r_s_ ≥ 0.4 or ≤−0.4) with flax type varied significantly between the studied groups of genes. For groups of *BGAL* genes and *ABC* plus *HMA* genes, a significant proportion (about 40%) of the studied genes had FTA polymorphisms. In groups of *CESA*, *TUB*, *CTL*, and *RGL* genes and genes involved in lignin synthesis, about 20–31% of the studied genes had polymorphisms with a strong correlation with flax type. In groups of fatty acid and lignan synthesis genes, fewer than 20% of the studied genes had FTA polymorphisms, while in the *ACT* group, there were no such genes. In general, these results were in concordance with those observed in the cluster analysis described above; the more polymorphisms with a high correlation coefficient in a group, the clearer the flax type-associated clusters formed for this group.

We also evaluated the expression levels of the studied genes (based on our and NCBI RNA-Seq data) with the focus on genes with polymorphisms with a strong correlation with flax type. The following types of flax tissues were included in the analysis: roots and shoots of seedlings, leaves, flowers, and stems of adult plants [74], capsules (obtained in the current study, PRJNA634481), cortical parenchyma (cPAR), intrusively growing fibers (iFIB) [43], fibers depositing tertiary cell wall (tFIB) [41,44], xylem part (sXYL) [44,45], and embryo (PRJNA720521). Data are presented in Supplementary Table S5 and Supplementary Figures S2 and S3 as heatmaps. Heatmaps revealed groups of genes that were predominantly expressed in particular flax tissues, suggesting their role in the formation of key characteristics of fiber flax or linseed. For example, genes with high expression levels in stem tissues could be crucial for fiber flax traits, while genes with high expression levels in capsules could be of paramount importance for linseed characteristics.

#### 2.2.2. Genes Involved in Lignin Synthesis

Among the genes related to lignin synthesis, *4CL1*, *4CL4*, *4CL5*, *C4H3*, *C4H4*, *CAD1A*, *CAD1B*, *CAD4A*, *CAD4B*, *CAD7*, *CCR11*, *CCR4*, *CCoAOMT5*, *COMT2*, *COMT3*, *F5H1*, *F5H7*, *PAL1*, and *PAL3* had polymorphisms with a strong correlation (r_s_ ≥ 0.4 or ≤−0.4) with flax type. For the majority of these genes, higher expression levels were observed in seedling roots, sXYL, and capsules. Within this group of genes, *PAL1* and *PAL3* had polymorphisms with a very strong correlation (r_s_ ≥ 0.6 or ≤−0.6) with flax type (1 and 2 polymorphisms respectively). *PAL1* was predominantly expressed in sXYL and could be associated with lignin synthesis in flax stems, while *PAL3* was predominantly expressed in seedling roots and could be associated with the root traits important for the formation of a particular flax plant type. We performed clustering based on the VAF of *PAL1* and *PAL3*; however, we did not reveal a clear enough association of clusters with flax type (Supplementary Figure S4a,b). This could be due to the fact that there are a significant number of various allelic variants containing these polymorphisms with a very strong correlation with flax type. *PAL* genes encode phenylalanine ammonia-lyases, which deaminate phenylalanine (the initial substrate in the lignin biosynthesis pathway) and result in cinnamic acid formation [75]. *PAL* is one of the key players in lignin synthesis [76], and downregulation or disruption of *PAL* results in a reduction in the lignin content and a change in the lignin composition in *Arabidopsis thaliana* [77], *Medicago sativa* [78] *Populus trichocarpa* [79], and *Nicotiana tabacum* [80,81].

The highest number (32) of polymorphisms with a strong correlation (r_s_ ≥ 0.4 or ≤−0.4) with flax type among the lignin synthesis genes was identified for *4CL1*. This gene was expressed more highly in seedling roots and sXYL than in other analyzed tissues. A cluster of predominantly linseed varieties and a cluster of predominantly fiber flax varieties were observed in the dendrogram based on the VAF of *4CL1* (Supplementary Figure S4c). *C4H4* also contained a significant number (9) of FTA polymorphisms. This gene was mostly expressed in seedling roots and sXYL. A cluster of predominantly linseed varieties, a cluster of predominantly fiber flax varieties, and a mixed cluster were observed based on the VAF of *C4H4* (Supplementary Figure S4d). A large number of FTA polymorphisms made a significant contribution to the separation of samples according to the flax type; however, the majority of these polymorphisms, most likely, do not affect the trait associated with the flax type but are inherited linked to a key polymorphism(s). It is known that *C4H* genes encode cinnamate 4-hydroxylases, which catalyze the second step in lignin biosynthesis (hydroxylation of cinnamic acid), while *4CL* genes, which encode 4-coumarate:CoA ligases, are responsible for the formation of *p*-coumaroyl CoA from *p*–coumaric acid [75]. *C4Hs* and *4CLs* along with *PALs* play an important role in lignin formation; the downregulation of *C4Hs* and *4CLs* was reported to decrease the lignin content [75,76,82].

Five *CAD* genes (*CAD1A*, *CAD1B*, *CAD4A*, *CAD4B*, and *CAD7*) of the thirteen studied had polymorphisms with a strong correlation (r_s_ ≥ 0.4 or ≤−0.4) with flax type. These five genes had increased expression levels in seedling roots; in addition, *CAD1A* and *CAD1B* were also highly expressed in sXYL, while *CAD4A* was also highly expressed in stems; these genes are of the most interest because they could be involved in the determination of the lignin content in flax stems. Among these three genes, clustering based on the VAF of *CAD1B* had the greatest concordance with flax type (Supplementary Figure S4e). *CAD* genes encode cinnamyl alcohol dehydrogenases, which catalyze the reduction of hydroxycinnamyl aldehydes into monolignols [56,75]. The downregulation of *CADs* resulted in changes in lignin composition in poplar [83] and cotton [84]. The brown-midrib phenotype of stems was revealed in flax *CAD* mutants [85]. In addition, the role of *CAD* genes in responses to stresses in plants, including flax, was also revealed [53,86,87,88,89].

The association of the polymorphisms of genes involved in the synthesis of lignin with flax type was likely due to the low content of lignin in the stem, which is an important characteristic of fiber flax. In addition, as alterations in lignin biosynthesis are implicated in the regulation of plant growth and defense [90], genes with FTA polymorphisms could be essential for stages of flax plant development that have different levels of importance for the formation of fiber flax and linseed yield and for responses to stresses that have a diverse impact on the products of linseed and fiber flax. Thus, expression analysis of genes with FTA polymorphisms could improve our understanding of which processes these genes are involved in.

#### 2.2.3. Cellulose Synthases

For genes encoding cellulose synthases, a strong correlation (r_s_ ≥ 0.4 or ≤−0.4) of polymorphisms with flax type was observed for *CESA1-B*, *CESA3-A*, *CESA4*, and *CESA8-A* (from one to two polymorphisms). These genes had increased expression levels in sXYL, and *CESA1-B* was also highly expressed in seedling roots. For *CESA1-B*, one polymorphism had a very strong correlation (r_s_ = −0.63) with flax type. Clustering based on the VAF of *CESA1-B* revealed a cluster of predominantly linseed varieties and a mixed cluster (Supplementary Figure S4f). *CESA1* and *CESA3* are involved in the cellulose synthesis of the primary cell wall, while *CESA4* and *CESA8* participate in the cellulose synthesis of the secondary cell wall [91]. Numerous studies on *CESA* genes indicated their role in flax stem formation [38,42,57,58,59,60,61]. Thus, the presence of FTA polymorphisms in *CESA1-B*, *CESA3-A*, *CESA4*, and *CESA8-A* is likely explained by their role in the determination of flax stem properties, which are especially important for fiber flax.

#### 2.2.4. Chitinase-Like Proteins

Among the genes encoding chitinase-like proteins, polymorphisms with a strong correlation (r_s_ ≥ 0.4 or ≤−0.4) with flax type were identified for *CTL1*, *CTL10*, *CTL13*, *CTL18*, *CTL2*, *CTL22*, *CTL23*, *CTL24*, *CTL26*, *CTL35*, and *CTL4.* The expression profiles of these genes in different tissues were quite dissimilar: *CTL1*, *CTL2*, and *CTL23* had increased expression levels in sXYL; *CTL23*, *CTL24*, *CTL26*, *CTL22*, and *CTL4* in capsules; *CTL13* and *CTL4* in seedling roots; and *CTL10* in leaves and flowers. Meanwhile, the expression of *CTL35* and *CTL18* was low in all analyzed tissues (CPM < 10 in each sample). The greatest number of FTA polymorphisms was revealed for *CTL1* and *CTL18*. In clustering based on the VAF of *CTL1*, a small cluster of predominantly linseed varieties and a large mixed cluster were observed (Supplementary Figure S4g). For *CTL18*, two clusters were revealed: in the first cluster, the majority of linseed varieties were included, and in the second one, fiber flax varieties prevailed. However, each cluster included a significant number of varieties of the other type (Supplementary Figure S4h). Considering that *CTL18* expression was low in all of the studied tissues, its role in the determination of traits associated with flax type is not clear, but it could be linked to a gene important for the formation of these traits. Chitinases are involved in plant stress response, development, and cell wall synthesis [92,93,94]. It was revealed that *CTL1* expression in flax was significantly higher in stem tissues in which thickening of the cell walls occurred, and coexpression of this gene and *CESA4*, *CESA7*, and *CESA8*, which are involved in the formation of the secondary cell wall, was observed, which could indicate the role of *CTL1* in flax cell wall synthesis [60]. Thus, *CTL1* is probably important for the formation of flax stem and, for this reason, polymorphisms of *CTL1* were associated with flax type.

#### 2.2.5. Tubulins

For tubulin-encoding genes, a strong correlation (r_s_ ≥ 0.4 or ≤−0.4) of polymorphisms with flax type was revealed for *Alfa_TUB2*, *Beta_TUB13*, *Beta_TUB3*, *Beta_TUB6*, and *Beta_TUB7*. All of these genes had increased expression levels in seedling roots and stem tissues. Several of these genes were also highly expressed in other tissue types, but there was a significant variation in such tissue types between genes. The greatest number (8) of FTA polymorphisms was identified for *Beta_TUB3*, and clustering based on the VAF of this gene had a relatively high concordance with flax type (Supplementary Figure S4i). Tubulins are involved in numerous processes in flax plants, including cell wall formation [59,64], and thus could influence traits that play an important role in the formation of stems. However, given the involvement of tubulins in numerous processes in plants, they also could participate in the formation of other traits that are important for fiber flax or linseed plants.

#### 2.2.6. β-Galactosidases

Among the 40 studied genes encoding β-galactosidase-like proteins, 16 had polymorphisms with a strong correlation (r_s_ ≥ 0.4 or ≤−0.4) with flax type (the number of such polymorphisms varied from 1 to 25). The expression profiles of these genes also varied; however, most genes had the highest expression levels in leaves, flowers, iFIB, and tFIB. *BGAL40* had the highest number (25) of FTA polymorphisms, and clustering of varieties based on the VAF of this gene was in high concordance with the division of varieties into fiber flax and linseed (Supplementary Figure S4j). This gene was predominantly expressed in leaves. For *BGAL30*, polymorphisms with a very strong correlation (r_s_ ≥ 0.6 or ≤−0.6) with flax type were revealed, and the highest expression level of this gene was observed in flowers. However, clustering based on the VAF of *BGAL30* did not show clear enough differentiation of clusters according to flax type (Supplementary Figure S4k); the influence of other polymorphisms was probably strong. It was shown that β-galactosidases are involved in the modification of cell wall polysaccharides [95,96]. In flax, β-galactosidases are important for the development of the secondary cell wall [58,97]; therefore, genes encoding β-galactosidase-like proteins are likely implicated in the formation of traits of flax stems, which are especially important for fiber flax, and, for this reason, we identified FTA polymorphisms in a large number of *BGALs*.

#### 2.2.7. Rhamnogalacturonan Lyases

Among rhamnogalacturonan lyases-encoding genes, *RGL1_B* and *RGL4_B* each had one polymorphism associated with flax type (r_s_ ≥ 0.4 or ≤−0.4). *RGL1_B* was predominantly expressed in sXYL, while the expression of *RGL4_B* was higher in leaves, flowers, and capsules; however, the expression level of this gene was also significant in all other studied tissues. It is known that rhamnogalacturonan lyases are involved in the modification of cell wall polysaccharides (RGLs degrade the rhamnogalacturonan I backbone) [98]. Transcriptomic studies of flax showed that rhamnogalacturonan lyases are probably involved in tertiary cell wall formation [41,57]. Based on gene expression data, it was suggested that *RGL1_B* plays role in the modification of cell wall polysaccharides in xylem tissues [57]. The identification of FTA polymorphism in *RGL1_B* could also indicate a role for this gene in flax cell wall formation and its importance for stem traits of fiber flax.

#### 2.2.8. Genes Involved in Lignan Synthesis

For genes related to lignan synthesis, *PLR1* had many FTA polymorphisms. This gene was predominantly expressed in capsules, and clustering based on the VAF of *PLR1* revealed clear enough differentiation of clusters according to flax type (Supplementary Figure S4l). In addition, one polymorphism in this gene had a very strong correlation (r_s_ = 0.64) with flax type. It was shown that *PLRs* are important for the synthesis of the major lignan in flax seeds, secoisolariciresinol diglucoside (SDG) [67,99]. SDG has antioxidative and anti-inflammatory capacities and reduces the risk of cancer and cardiac diseases [8]. PLR1 contributes to the synthesis of (+)-secoisolariciresinol by catalyzing the conversion of (−)-pinoresinol into (−)-lariciresinol and is necessary for the accumulation of SDG in flax seeds [67,100,101]. In addition, the implication of *PLR1* in the flax plant stress response was revealed [102]. Thus, a large number of FTA polymorphisms of *PLR1* could be due to its importance for the formation of a linseed trait, the lignan content. However, the difference in linseed and fiber flax based on *PLR1* polymorphisms could also be associated with the role of this gene in the formation of other characteristics, which are important for a particular flax type, namely, stress response.

#### 2.2.9. Genes Involved in Fatty Acid Synthesis

Among the studied genes related to fatty acid synthesis, *FAD2A*, encoding fatty acid desaturases 2, had one polymorphism associated with flax type (r_s_ = −0.44). The expression of *FAD2A* was higher in capsules and embryo than in other analyzed tissues, and clustering had some concordance with flax type, but it was poor enough (Supplementary Figure S4m). It is known that FAD2 catalyzes the desaturation of oleic acid into linoleic acid, while FAD3 catalyzes linoleic acid into linolenic acid, and polymorphisms of *FAD* genes are associated with the content of these fatty acids, which was especially clearly shown for *FAD3A* and *FAD3B* genes [34,71,103,104,105]. However, the present study did not reveal an association between flax type and polymorphisms in *FAD3A* and *FAD3B* genes. This may be due to the fact that most linseed varieties similar to fiber ones did not carry the key polymorphisms in *FAD3* genes that determine a lower content of linolenic acid [106], and the used sample set did not include a significant number of low-linolenic varieties that could form a separate cluster.

#### 2.2.10. ABC Transporter and Heavy Metal-Associated Genes

Within the studied ABC transporter and heavy metal-associated genes, 83 genes had polymorphisms with a strong correlation (r_s_ ≥ 0.4 or ≤−0.4) with flax type, and the number of such polymorphisms per gene varied from 1 to 39. The expression profiles of these genes in the studied tissues were very different, but in general, most genes had the highest expression levels in seedling roots. For *ABCA1*, *ABCA7*, *ABCB42*, *ABCB47*, *ABCG71*, and *ABCG80* genes with a high number (27–39) of FTA polymorphisms, VAF clustering revealed a linseed cluster, whose size varied from gene to gene, while the rest clusters were not clear enough differentiated according to flax type (Supplementary Figure S4n,o–q,s), except for *ABCG71*, for which two clusters of predominantly linseed or fiber flax varieties were revealed (Supplementary Figure S4r). For *ABCG79*, *ABCB42*, *ABCH1*, *ABCB40*, *ABCB45*, *ABCB47*, *HMA12*, *ABCG8*, and *ABCC4* genes, polymorphisms with a very strong correlation (r_s_ ≥ 0.6 or ≤−0.6) with flax type were identified, and the expression profiles of these genes in the studied flax tissues varied greatly. The highest expression level of *ABCG79* was revealed in leaves and seedling shoots; *ABCB42* in seedling roots; *ABCH1* in leaves, capsules, seedling shoots, iFIB, and tFIB; *ABCB40* in flowers; *ABCB45* in seedling roots and sXYL; *HMA12* in sXYL; *ABCG8* in seedling shoots; and *ABCC4* in iFIB. ABC transporters are essential components of plant cell membranes and are involved in numerous processes, including growth and development, nutrition, and responses to abiotic and biotic stresses [107]. Using a general linear model (GLM), it was shown that the *Lus10016125* gene (*ABCG4* according to the classification by Khan et al. [73]) is probably involved in the determination of flax plant height [31]. In the present study, three polymorphisms of *ABCG4* had a strong association with flax type, but other *ABC* and *HMA* genes had more FTA polymorphisms or polymorphisms with a very strong correlation (r_s_ ≥ 0.6 or ≤−0.6) with flax type. Among our analyzed groups of genes, the group of *ABC* and *HMA* genes had one of the highest proportions of genes with FTA polymorphisms (40%). As the functions of *ABC* and *HMA* genes vary and their expression profiles were diverse between flax tissues, they could be involved in the determination of traits of different organs of flax plants, which ultimately define features that are important for linseed or fiber flax.

## 3. Discussion

Linseed and fiber flax differ not only in the characteristics of plant parts that are key for agricultural use, namely, the seeds and stems, but also in the anatomy and morphology of the roots (in linseed varieties, in comparison with fiber flax ones, roots penetrate to a greater depth, lateral roots are thicker and longer, the root system has a large absorption area, more distribution to a greater depth, and more developed conducting system), as well as the consumption of nutrients in different periods of plant development (for fiber flax, the critical periods of nutrition correspond to the initial periods of vegetation development, while for linseed, the critical periods are dissimilar and later stages of development are also important), the requirements for the content of nutrients in soil (linseed has higher requirements for nitrogen and phosphorus, whereas fiber flax has a higher requirement for potassium), and the ability to grow under unfavorable conditions (unlike fiber flax, it is more significant for linseed to grow in soils with a higher salt content than in acidic soils) [108]. Therefore, for linseed and fiber flax, different stages of development are critical for the formation of seeds and fiber, respectively, and diverse stresses affect these flax plants in dissimilar ways. In this regard, an important role in the formation of fiber and oil flax plants capable of producing high yields of fiber and seeds, respectively, is probably played by different genes, some of which ensure the optimal development of linseed, while others, fiber flax, due to the distinctive features that are typical for two types of flax plants. Thus, some genes apparently play a role in the formation of stems and seeds, and some genes contribute to the formation of other plant traits, for example, the root system or resistance to specific unfavorable environments at particular stages of development, which indirectly affect the ability of flax plants to produce high levels of high-quality fiber or seeds. Likely, when linseed breeding took place, there was a selection for some alleles of these two groups of genes, and during the breeding of fiber flax, other alleles were preferred since these genes had a different significance for the formation of a flax type with favorable economically valuable traits. Thus, an approach based on the search for genes whose polymorphisms are associated with the flax plant type can provide new knowledge about genes that are important for the development of linseed or fiber flax and, therefore, affect the quality of flax production. Such genes can be used in genomic breeding and marker-assisted selection of flax.

Various approaches are used for the identification of genetic markers or genes associated with important flax plant characteristics. Linkage mapping and association mapping/genome-wide association studies (GWAS) have allowed scientists to identify quantitative trait loci (QTL) and quantitative trait nucleotides (QTN) associated with seed yield and quality traits, fiber traits, agronomic traits, disease resistance, and abiotic stress response in flax [29,31,32,33,34,35,36,109,110,111,112,113,114,115,116]. In addition to GWAS, transcriptome analysis can be useful to avoid the false-positive results in candidate gene searches; using such a combined approach, candidate genes related to fatty acid synthesis in flax seeds were identified [37]. Another approach that was successfully used for the identification of fiber quality-associated genes was based on the analysis of gene expression in different tissues of flax to reveal genes with tissue-specific expression in developing fibers and their further analysis [38]. Such an analysis is valuable if there are transcriptomic data for the specific tissues at certain stages of plant development, and it would be preferable if there were data for several genotypes, which are more difficult to obtain. In addition, the appearance of new data (additional tissues or genotypes) may change the results of the analysis. In the present work, we focused on the study of particular gene families, whose role in plant growth, development, and stress response is known and which were identified in the flax genome. First, we searched for genes with polymorphisms associated with the flax plant type based on the data of whole-genome sequencing of a large number of varieties and then analyzed the expression profiles of these genes in various flax tissues. Such an approach allowed us to use the currently available data as efficiently as possible and narrow the range of analysis to families of sufficiently characterized functional genes in order to conduct a more detailed analysis of their possible role in the determination of traits that have different levels of significance for the formation of fiber flax or linseed plants. Genes, the polymorphisms of which had a strong association with the flax plant type, were probably subjected to selection pressure; the alleles that were more common in oil flax differed from those that were characteristic of fiber flax. Moreover, such genes can determine not only the traits of flax seeds and stems but also, for example, the characteristics of the root system or resistance of flax plants to specific stresses at a particular stage of development, which indirectly affect the ability of plants to produce seed or fiber.

## 4. Materials and Methods

### 4.1. Variant Calling and Associative Analysis of Genome Sequencing Data

For association analysis between allele variants and flax type (fiber flax or linseed), we used Illumina whole-genome sequencing (WGS) data from two NCBI BioProjects: PRJNA590636 (12× average coverage; [36]) and PRJNA478805 (25× average coverage). In total, 191 flax varieties (79 fiber flax and 112 linseed cultivars/lines) were analyzed. The list of varieties can be found in Supplementary Tables S1, S3 and S4. Downloaded reads were trimmed and adapters were removed with Trimmomatic 0.38 [117]. Then, reads were mapped to the reference *L. usitatissimum* genome assembly GCA_000224295.2 (ASM22429v2) by BWA-MEM 0.7.17 [118] with lowered minimum seed length (‘-k’ argument). Subsequently, sorted BAM files were processed with FixMateInformation (picard-tools 2.21.3) (http://broadinstitute.github.io/picard/, accessed on 27 October 2021). Next, duplicated reads were marked with MarkDuplicatesWithMateCigar (picard-tools). Finally, variant calling was performed by freeBayes 1.3.2 [119] for the joint set of 191 BAM files with the following thresholds: mapping quality 10, base calling quality 15, and minimal alternative allele coverage 4 (maximal value across all samples). The following genes were analyzed: 66 genes related to lignin synthesis—*4CL1*, *4CL2*, *4CL3*, *4CL4*, *4CL5*, *4CL6*, *4CL7*, *4CL8*, *4CL9*, *C3′H1*, *C3′H2*, *C3′H3*, *C4H1*, *C4H2*, *C4H3*, *C4H4*, *C4H5*, *CAD1A*, *CAD1B*, *CAD2A*, *CAD2B*, *CAD3A*, *CAD3B*, *CAD4A*, *CAD4B*, *CAD5A*, *CAD5B*, *CAD6*, *CAD7*, *CAD8*, *CCoAOMT1*, *CCoAOMT2*, *CCoAOMT3*, *CCoAOMT4*, *CCoAOMT5*, *CCR1*, *CCR10*, *CCR11*, *CCR12*, *CCR2*, *CCR3*, *CCR4*, *CCR5*, *CCR6*, *CCR7*, *CCR8*, *CCR9*, *COMT1*, *COMT2*, *COMT3*, *F5H1*, *F5H2*, *F5H3*, *F5H4*, *F5H5*, *F5H6*, *F5H7*, *F5H8*, *HCT1*, *HCT2*, *HCT3*, *HCT4*, *HCT5*, *PAL1*, *PAL2*, and *PAL3*; 35 genes encoding chitinase-like proteins—*CTL1*, *CTL10*, *CTL11*, *CTL12*, *CTL13*, *CTL14*, *CTL16*, *CTL17*, *CTL18*, *CTL19*, *CTL2*, *CTL20*, *CTL21*, *CTL22*, *CTL23*, *CTL24*, *CTL25*, *CTL26*, *CTL27*, *CTL28*, *CTL29*, *CTL3*, *CTL30*, *CTL31*, *CTL32*, *CTL33*, *CTL35*, *CTL36*, *CTL37*, *CTL4*, *CTL5*, *CTL6*, *CTL7*, *CTL8*, and *CTL9*; 40 genes encoding β-galactosidases—*BGAL1*, *BGAL10*, *BGAL11*, *BGAL12*, *BGAL13*, *BGAL14*, *BGAL16*, *BGAL18*, *BGAL19*, *BGAL2*, *BGAL20*, *BGAL21*, *BGAL22*, *BGAL23*, *BGAL24*, *BGAL25*, *BGAL26*, *BGAL27*, *BGAL28*, *BGAL29*, *BGAL3*, *BGAL30*, *BGAL31*, *BGAL32*, *BGAL33*, *BGAL34*, *BGAL35*, *BGAL36*, *BGAL37*, *BGAL38*, *BGAL39*, *BGAL4*, *BGAL40*, *BGAL41*, *BGAL42*, *BGAL43*, *BGAL6*, *BGAL7*, *BGAL8*, and *BGAL9*; 206 genes encoding ABC transporters and heavy metal–associated genes*—ABCA1*, *ABCA2*, *ABCA3*, *ABCA4*, *ABCA5*, *ABCA6*, *ABCA7*, *ABCA8*, *ABCB1*, *ABCB10*, *ABCB11*, *ABCB12*, *ABCB13*, *ABCB14*, *ABCB15*, *ABCB16*, *ABCB17*, *ABCB18*, *ABCB19*, *ABCB2*, *ABCB20*, *ABCB21*, *ABCB22*, *ABCB23*, *ABCB24*, *ABCB25*, *ABCB26*, *ABCB27*, *ABCB28*, *ABCB29*, *ABCB3*, *ABCB30*, *ABCB31*, *ABCB32*, *ABCB33*, *ABCB34*, *ABCB35*, *ABCB36*, *ABCB37*, *ABCB38*, *ABCB39*, *ABCB4*, *ABCB40*, *ABCB41*, *ABCB42*, *ABCB43*, *ABCB44*, *ABCB45*, *ABCB46*, *ABCB47*, *ABCB48*, *ABCB5*, *ABCB6*, *ABCB7*, *ABCB8*, *ABCB9*, *ABCC1*, *ABCC10*, *ABCC11*, *ABCC12*, *ABCC13*, *ABCC14*, *ABCC15*, *ABCC16*, *ABCC17*, *ABCC18*, *ABCC19*, *ABCC2*, *ABCC3*, *ABCC4*, *ABCC5*, *ABCC6*, *ABCC7*, *ABCC8*, *ABCC9*, *ABCD1*, *ABCD2*, *ABCD3*, *ABCD4*, *ABCD5*, *ABCE1*, *ABCE2*, *ABCF1*, *ABCF2*, *ABCF3*, *ABCF4*, *ABCF5*, *ABCF6*, *ABCF7*, *ABCF8*, *ABCF9*, *ABCG1*, *ABCG11*, *ABCG12*, *ABCG13*, *ABCG14*, *ABCG16*, *ABCG17*, *ABCG18*, *ABCG19*, *ABCG2*, *ABCG20*, *ABCG21*, *ABCG22*, *ABCG23*, *ABCG24*, *ABCG25*, *ABCG26*, *ABCG27*, *ABCG28*, *ABCG29*, *ABCG3*, *ABCG30*, *ABCG31*, *ABCG32*, *ABCG33*, *ABCG34*, *ABCG35*, *ABCG36*, *ABCG37*, *ABCG38*, *ABCG39*, *ABCG4*, *ABCG40*, *ABCG41*, *ABCG42*, *ABCG43*, *ABCG44*, *ABCG45*, *ABCG46*, *ABCG47*, *ABCG48*, *ABCG49*, *ABCG5*, *ABCG50*, *ABCG51*, *ABCG52*, *ABCG53*, *ABCG54*, *ABCG55*, *ABCG56*, *ABCG57*, *ABCG58*, *ABCG59*, *ABCG6*, *ABCG60*, *ABCG61*, *ABCG62*, *ABCG63*, *ABCG64*, *ABCG65*, *ABCG66*, *ABCG67*, *ABCG68*, *ABCG69*, *ABCG7*, *ABCG70*, *ABCG71*, *ABCG72*, *ABCG73*, *ABCG74*, *ABCG75*, *ABCG76*, *ABCG77*, *ABCG78*, *ABCG79*, *ABCG8*, *ABCG80*, *ABCG81*, *ABCG82*, *ABCG83*, *ABCG84*, *ABCG85*, *ABCG9*, *ABCH1*, *ABCH10*, *ABCH11*, *ABCH12*, *ABCH13*, *ABCH14*, *ABCH15*, *ABCH16*, *ABCH17*, *ABCH18*, *ABCH19*, *ABCH2*, *ABCH20*, *ABCH21*, *ABCH22*, *ABCH3*, *ABCH4*, *ABCH5*, *ABCH6*, *ABCH7*, *ABCH8*, *HMA1*, *HMA10*, *HMA11*, *HMA12*, *HMA2*, *HMA3*, *HMA4*, *HMA6*, *HMA7*, *HMA8*, and *HMA9*; 9 genes related to lignan synthesis—*DIR1*, *DIR2*, *DIR3*, *DIR4*, *DIR5*, *DIR6*, *PLR1*, *PLR2*, and *UGT74S1*; 21 genes encoding tubulins—*Alfa_TUB1*, *Alfa_TUB2*, *Alfa_TUB3*, *Alfa_TUB4*, *Alfa_TUB5*, *Alfa_TUB6*, *Beta_TUB1*, *Beta_TUB10*, *Beta_TUB11*, *Beta_TUB12*, *Beta_TUB13*, *Beta_TUB2*, *Beta_TUB3*, *Beta_TUB4*, *Beta_TUB5*, *Beta_TUB6*, *Beta_TUB7*, *Beta_TUB8*, *Beta_TUB9*, *Gamma_Tub1*, and *Gamma_Tub2*; 16 genes encoding cellulose synthases—*CESA1-A*, *CESA1-B*, *CESA3-A*, *CESA3-B*, *CESA3-C*, *CESA4*, *CESA6-A*, *CESA6-B*, *CESA6-C*, *CESA6-D*, *CESA6-E*, *CESA6-F*, *CESA7-A*, *CESA7-B*, *CESA8-A*, and *CESA8-B*; 10 genes encoding rhamnogalacturonate lyases—*RGL1_B*, *RGL2*, *RGL3_A*, *RGL3_B*, *RGL4_A*, *RGL4_B*, *RGL6_A*, *RGL6_B*, *RGL7_A*, and *RGL7_B*; 6 genes related to fatty acid synthesis—*FAD2A*, *FAD2B*, *FAD3A*, *FAD3B*, *SAD1*, and *SAD2*; and 15 genes encoding actins—*Act1*, *Act10*, *Act11*, *Act12*, *Act13*, *Act14*, *Act15*, *Act2*, *Act3*, *Act4*, *Act5*, *Act6*, *Act7*, *Act8*, and *Act9.* Data for gene sequences/genome coordinates were obtained from the literature [38,53,56,57,58,60,64,65,73,105,120]. Analyzed genome regions included the gene body, 500 bp upstream, and 500 bp downstream. To evaluate the number of polymorphisms per gene, we counted point substitutions and indels that were supported with at least four reads (regardless of variant allele frequency, VAF) and were observed in at least one sample. Such thresholds were chosen because of the low overall genome coverage (only 7× after marking duplicates) for the analyzed samples from NCBI BioProjects PRJNA590636 and PRJNA478805.

Next, we performed correlation analysis between the VAF values and whether a variety belonged to linseed or fiber flax. Spearman’s, Pearson’s, and Kendall’s correlation coefficients and *p*-values were calculated. Additionally, VAF values were compared between these two groups using the nonparametric Mann–Whitney U-test. The derived *p*-values were adjusted for multiple testing with the Benjamini–Hochberg approach. Moreover, based on the similarity of the VAF profiles across variants (Euclidean distance), we performed hierarchical clustering of varieties using Ward’s minimum variance method (‘ward.D2’ in R 3.6.2).

### 4.2. Analysis of Transcriptome Sequencing Data

For gene expression analysis, we used NCBI BioProjects PRJNA475325 (RNA-Seq analysis of intrusively growing fibers from the flax stem) [43], PRJNA631357 (RNA-Seq analysis of phloem fibers during gravitropic behavior of flax plants) [45], PRJNA720521 (RNA-Seq flax data for the embryo and endosperm), PRJNA663265 (RNA-Seq dataset of multiple organs from flax), and PRJNA634481 (roots and shoots of seedlings and leaves, flowers, and stems of adult plants of six flax varieties; our data published in the data report format) [74]. In addition, our novel data for flax capsules were also used in the present study. In brief, seven flax varieties (AGT 409/10, AGT 427/10, AGT 981/05, AGT 1568/07, Atlant, LM 98, and Lola) were grown in a greenhouse, and capsules were harvested in the stage of yellow–green ripening. RNA for each variety was extracted from a pool of five capsules using a Quick-RNA Miniprep Kit (Zymo Research, Irvine, CA, USA), cDNA library preparation was carried out with NEBNext Ultra II Directional RNA Library Prep Kit for Illumina (New England Biolabs, Hertfordshire, UK), and sequencing was performed on NextSeq 500 (Illumina, San Diego, CA, USA) with a read length of 86 bp, as described in our previous study [74].

In total, 69 samples were included in the analysis. To eliminate mapping bias, reads from all samples were cropped to the minimal length across all samples (86 nt) with Trimmomatic. When paired-end read data were available, only forward read data were used. Next, reads were mapped to the reference *L. usitatissimum* genome assembly GCA_000224295.2 (ASM22429v2) with splice-aware STAR 2.7.2b mapper [121]. Since this reference assembly was not annotated, we relied only on de novo splice junction discovery and launched STAR in 2-pass mode. After the first pass, we collected all found splice junctions from all the samples and supplied this list for the second STAR pass.

The derived BAM files were sorted with samtools 1.10 and reordered, and read groups were assigned with picard-tools. Then, reads that contained Ns in their CIGAR field (e.g., spanning splicing junctions) were split with SplitNCigarReads from GATK 4.2.2.0 [122]. Next, read counts “per gene” were evaluated with bedtools multcov 2.26.0. We used a BED file containing the list of 424 regions containing genes, which were also applied in the search for FTA polymorphisms. We used preliminary splitting reads by splice junctions (with SplitNCigarReads tool) to exclude from counting those reads that, without mapping themselves to the region of interest (from the BED file), cross this region “as an intron”, with their first half mapping upstream of the region and the second half mapping downstream of the region. There were many such reads. The derived read counts were analyzed in edgeR [123] and normalized by the total number of mapped reads per sample. Heatmaps of expression levels of the studied genes in different flax tissues were created in the R environment using the pheatmap package. Expression values were log2-transformed and then normalized to the average value across all the samples (per each gene). Hierarchical clustering of genes and tissues was done using Ward’s minimum variance method (‘ward.D2’).

## Figures and Tables

**Table 1:** Number of flax type-associated polymorphisms (r_s_ ≥ 0.4 or ≤−0.4) for genes of the *4CL*, *C4H*, *CAD*, *CCR*, *CCoAOMT*, *COMT*, *F5H*, *PAL*, *CTL*, *BGAL*, *ABC*, *HMA*, *PLR*, *TUB*, *CESA*, *RGL*, and *FAD* families.

Gene	Number of FTA Polymorphisms	Gene	Number of FTA Polymorphisms	Gene	Number of FTA Polymorphisms	Gene	Number of FTA Polymorphisms
**Lignin synthesis**		*BGAL30*	21	*ABCB45*	19	*ABCG68*	2
*4CL1*	32	*BGAL31*	1	*ABCB46*	7	*ABCG69*	4
*4CL4*	2	*BGAL32*	4	*ABCB47*	30	*ABCG71*	30
*4CL5*	3	*BGAL33*	6	*ABCB48*	5	*ABCG72*	2
*C4H3*	2	*BGAL35*	1	*ABCB7*	6	*ABCG73*	15
*C4H4*	9	*BGAL37*	7	*ABCC10*	19	*ABCG75*	1
*CAD1A*	1	*BGAL40*	25	*ABCC16*	1	*ABCG79*	15
*CAD1B*	2	*BGAL41*	1	*ABCC18*	2	*ABCG8*	19
*CAD4A*	1	*BGAL6*	12	*ABCC4*	5	*ABCG80*	39
*CAD4B*	4	*BGAL7*	4	*ABCC6*	1	*ABCG83*	10
*CAD7*	2	*BGAL9*	4	*ABCC5*	1	*ABCH1*	3
*CCR11*	1	***ABC*** ** and *HMA***		*ABCF8*	2	*ABCH10*	15
*CCR4*	3	*ABCA1*	32	*ABCG1*	2	*ABCH11*	4
*CCoAOMT5*	2	*ABCA2*	16	*ABCG11*	3	*ABCH12*	1
*COMT2*	5	*ABCA3*	1	*ABCG12*	2	*ABCH8*	1
*COMT3*	2	*ABCA4*	1	*ABCG13*	3	*HMA12*	18
*F5H1*	2	*ABCA5*	11	*ABCG14*	8	*HMA2*	2
*F5H7*	1	*ABCA6*	2	*ABCG16*	15	*HMA3*	2
*PAL1*	2	*ABCA7*	27	*ABCG22*	6	*HMA4*	9
*PAL3*	3	*ABCA8*	12	*ABCG24*	1	*HMA6*	13
***CTL***		*ABCB1*	3	*ABCG25*	7	**Lignan synthesis**	
*CTL1*	16	*ABCB12*	1	*ABCG33*	4	*PLR1*	50
*CTL10*	6	*ABCB13*	10	*ABCG35*	15	***TUB***	
*CTL13*	8	*ABCB16*	2	*ABCG36*	4	*Alfa_TUB2*	1
*CTL18*	11	*ABCB2*	1	*ABCG37*	1	*Beta_TUB13*	1
*CTL2*	7	*ABCB22*	2	*ABCG4*	3	*Beta_TUB3*	8
*CTL22*	*1*	*ABCB23*	1	*ABCG40*	1	*Beta_TUB6*	6
*CTL23*	*7*	*ABCB25*	1	*ABCG47*	8	*Beta_TUB7*	2
*CTL24*	*3*	*ABCB26*	1	*ABCG52*	2	***CESA***	
*CTL26*	2	*ABCB29*	23	*ABCG56*	13	*CESA1-B*	1
*CTL35*	2	*ABCB3*	2	*ABCG57*	2	*CESA3-A*	1
*CTL4*	1	*ABCB32*	2	*ABCG58*	12	*CESA4*	2
***BGAL***		*ABCB33*	3	*ABCG59*	1	*CESA8-A*	1
*BGAL1*	2	*ABCB37*	1	*ABCG6*	4	***RGL***	
*BGAL10*	1	*ABCB39*	1	*ABCG60*	2	*RGL1_B*	1
*BGAL2*	11	*ABCB40*	22	*ABCG61*	1	*RGL4_B*	1
*BGAL23*	1	*ABCB42*	27	*ABCG62*	2	**Fatty acid synthesis**	
*BGAL27*	13	*ABCB43*	2	*ABCG64*	4	*FAD2A*	1

Note: If no FTA (flax type-associated) polymorphisms were identified for a particular gene, this gene was not presented in the table.

## Data Availability

The raw sequencing data have been deposited in the NCBI Sequence Read Archive (SRA) under the BioProject accession number PRJNA634481.

## Supplementary Materials

The following are available online at https://www.mdpi.com/article/10.3390/plants10122616/s1, Figure S1: Clustering of linseed and fiber flax varieties based on polymorphisms in lignin-associated (*4CL*, *C3′H*, *C4H*, *CAD*, *CCR*, *CCoAOMT*, *COMT*, *F5H*, *HCT*, and *PAL*) (a), *CTL* (b), *BGAL* (c), *ABC* and *HMA* (d), lignan-associated (*DIR*, *PLR*, and *UGT)* (e), *TUB* (f), *CESA* (g), *RGL* (h), fatty acid-associated (*FAD* and *SAD*) (i), and *ACT* (j) gene families; Figure S2: Heatmap of expression levels of genes of *4CL*, *C3′H*, *C4H*, *CAD*, *CCR*, *CCoAOMT*, *COMT*, *F5H*, *HCT*, *PAL*, *CTL*, *BGAL*, *ABC*, *HMA*, *DIR*, *PLR*, *UGT*, *TUB*, *CESA*, *RGL*, *FAD*, *SAD*, and *ACT* families in different flax tissues (averaged by tissue type); Figure S3: Heatmap of expression levels of genes of *4CL*, *C3′H*, *C4H*, *CAD*, *CCR*, *CCoAOMT*, *COMT*, *F5H*, *HCT*, *PAL*, *CTL*, *BGAL*, *ABC*, *HMA*, *DIR*, *PLR*, *UGT*, *TUB*, *CESA*, *RGL*, *FAD*, *SAD*, and *ACT* families in different flax tissues (individual samples); Figure S4: Clustering of linseed and fiber flax varieties based on polymorphisms in *PAL1* (a), *PAL3* (b), *4CL1* (c), *C4H4* (d), *CAD1B* (e), *CESA1-B* (f), *CTL1* (g), *CTL18* (h), *Beta_TUB3* (i), *BGAL40* (j), *BGAL30* (k), *PLR1* (l), *FAD2A* (m), *ABCA1* (n), *ABCA7* (o), *ABCB42* (p), *ABCB47* (q), *ABCG71* (r), and *ABCG80* (s) genes; Table S1: Polymorphisms in the studied genes of *4CL*, *C3′H*, *C4H*, *CAD*, *CCR*, *CCoAOMT*, *COMT*, *F5H*, *HCT*, *PAL*, *CTL*, *BGAL*, *ABC*, *HMA*, *DIR*, *PLR*, *UGT*, *TUB*, *CESA*, *RGL*, *FAD*, *SAD*, and *ACT* families in 191 flax varieties; Table S2: Data on the number of polymorphisms for individual genes of *4CL*, *C3′H*, *C4H*, *CAD*, *CCR*, *CCoAOMT*, *COMT*, *F5H*, *HCT*, *PAL*, *CTL*, *BGAL*, *ABC*, *HMA*, *DIR*, *PLR*, *UGT*, *TUB*, *CESA*, *RGL*, *FAD*, *SAD*, and *ACT* families taking into account the length of the analyzed region (gene length + 1000 bp, 500 bp upstream and 500 bp downstream); Table S3: Genetic similarity between fiber flax and linseed varieties based on polymorphisms in the studied genes of *4CL*, *C3′H*, *C4H*, *CAD*, *CCR*, *CCoAOMT*, *COMT*, *F5H*, *HCT*, *PAL*, *CTL*, *BGAL*, *ABC*, *HMA*, *DIR*, *PLR*, *UGT*, *TUB*, *CESA*, *RGL*, *FAD*, *SAD*, and *ACT* families; Table S4: Polymorphisms in the genes of *4CL*, *C4H*, *CAD*, *CCR*, *CCoAOMT*, *COMT*, *F5H*, *PAL*, *CTL*, *BGAL*, *ABC*, *HMA*, *PLR*, *TUB*, *CESA*, *RGL*, and *FAD* families with a strong correlation (r_s_ ≥ 0.4 or ≤−0.4) with flax type; Table S5: Expression levels of the genes of *4CL*, *C3′H*, *C4H*, *CAD*, *CCR*, *CCoAOMT*, *COMT*, *F5H*, *HCT*, *PAL*, *CTL*, *BGAL*, *ABC*, *HMA*, *DIR*, *PLR*, *UGT*, *TUB*, *CESA*, *RGL*, *FAD*, *SAD*, and *ACT* families in different flax tissues.
