# Supplementary material for: Genes Associated with the Flax Plant Type (Oil or Fiber) Identified Based on Genome and Transcriptome Sequencing Data
Source: Plants (Basel). 2021 Nov 28;10(12):2616. doi: 10.3390/plants10122616 (PMC8707629; doi:10.3390/plants10122616)

Supplementary Figure S1a. Dendrogram of linseed and fiber flax varieties based on polymorphisms of genes involved in lignin synthesis (*4CL*, *C3'H*, *C4H*, *CAD*, *CCR*, *CCoAOMT*, *COMT*, *F5H*, *HCT*, and *PAL* families). Linseed varieties are marked in blue and fiber flax ones are marked in red.

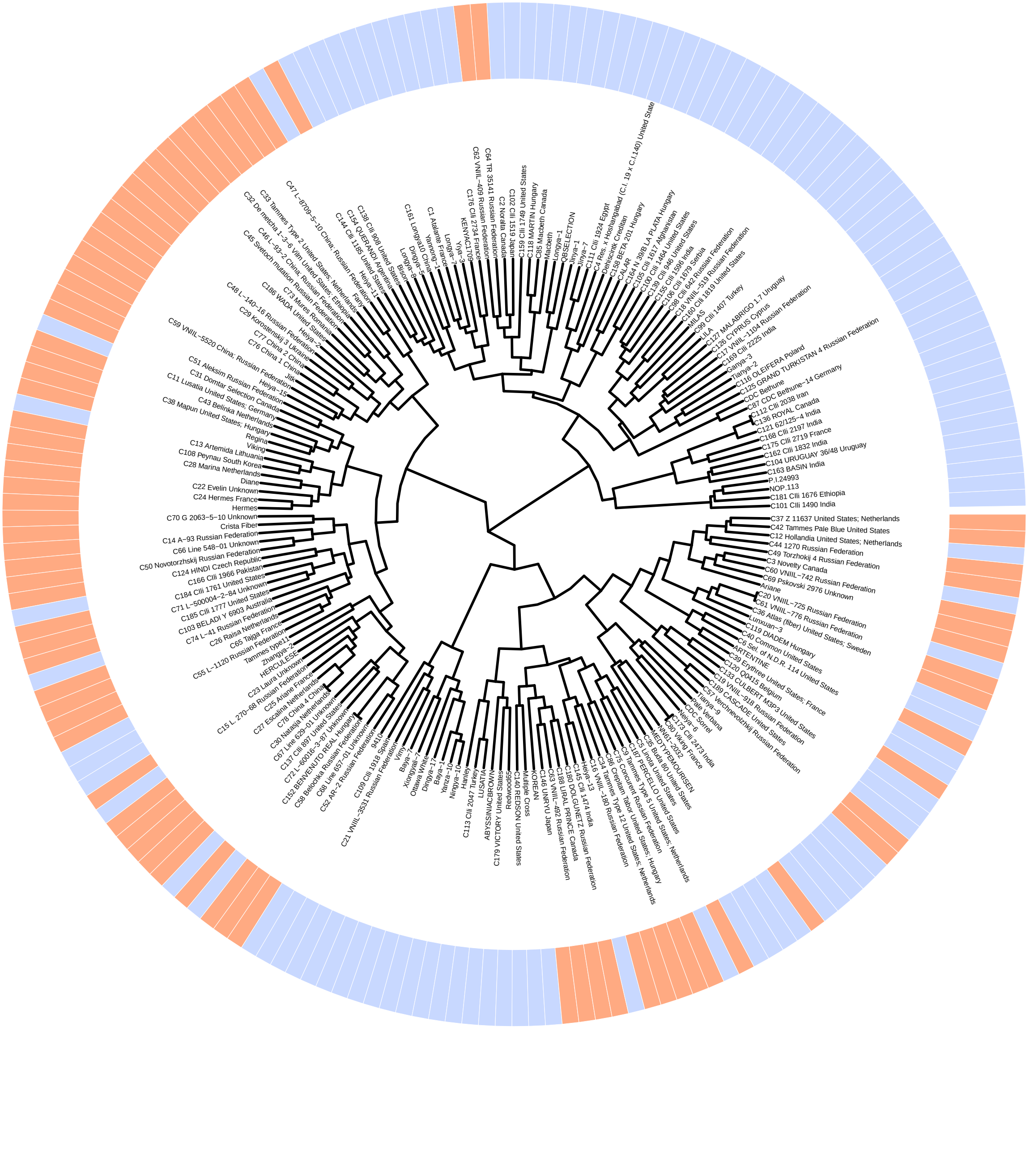

Supplementary Figure S1b. Dendrogram of linseed and fiber flax varieties based on polymorphisms of *CTL* genes.

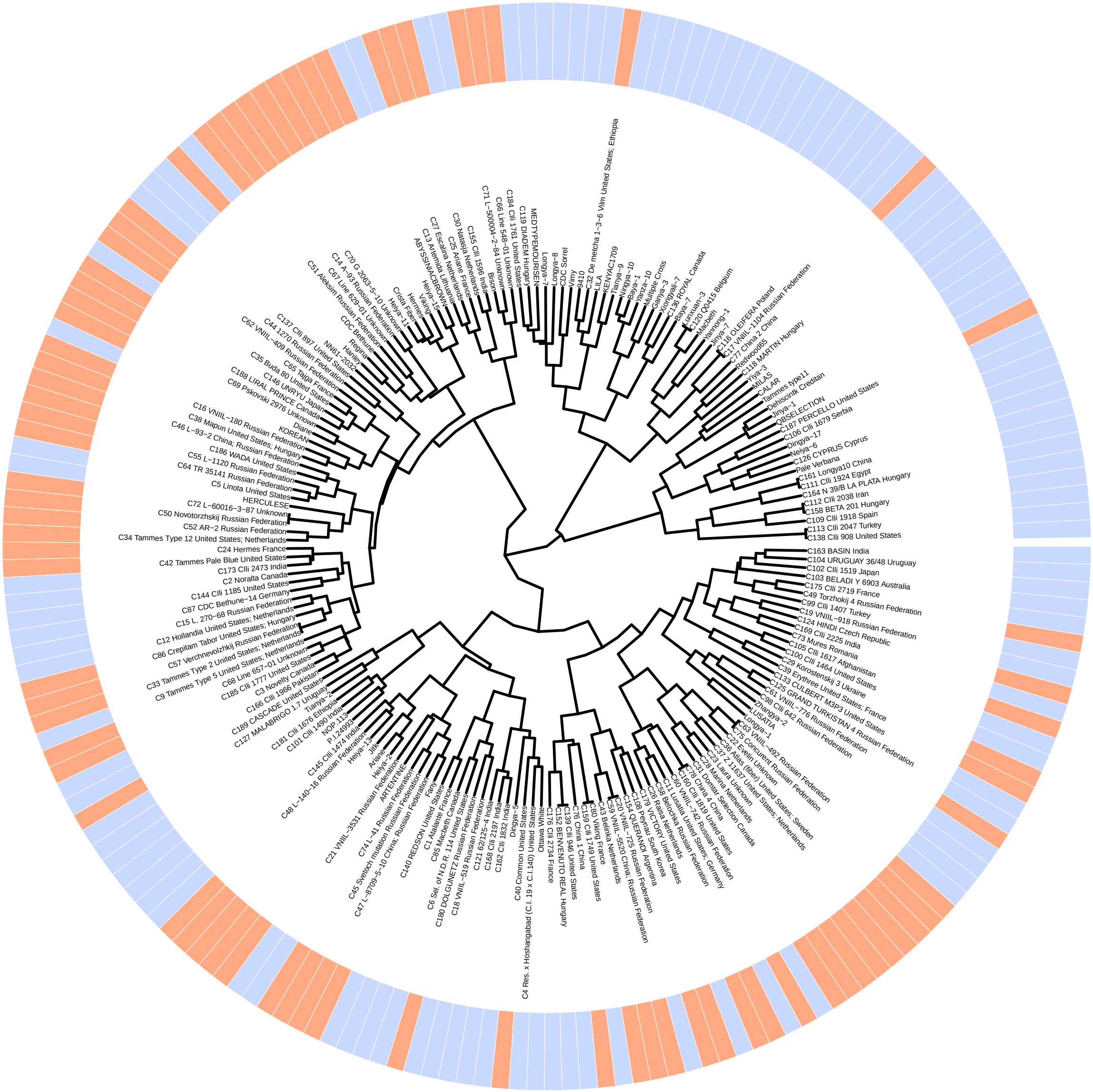

Supplementary Figure S1c. Dendrogram of linseed and fiber flax varieties based on polymorphisms of *BGAL* genes.

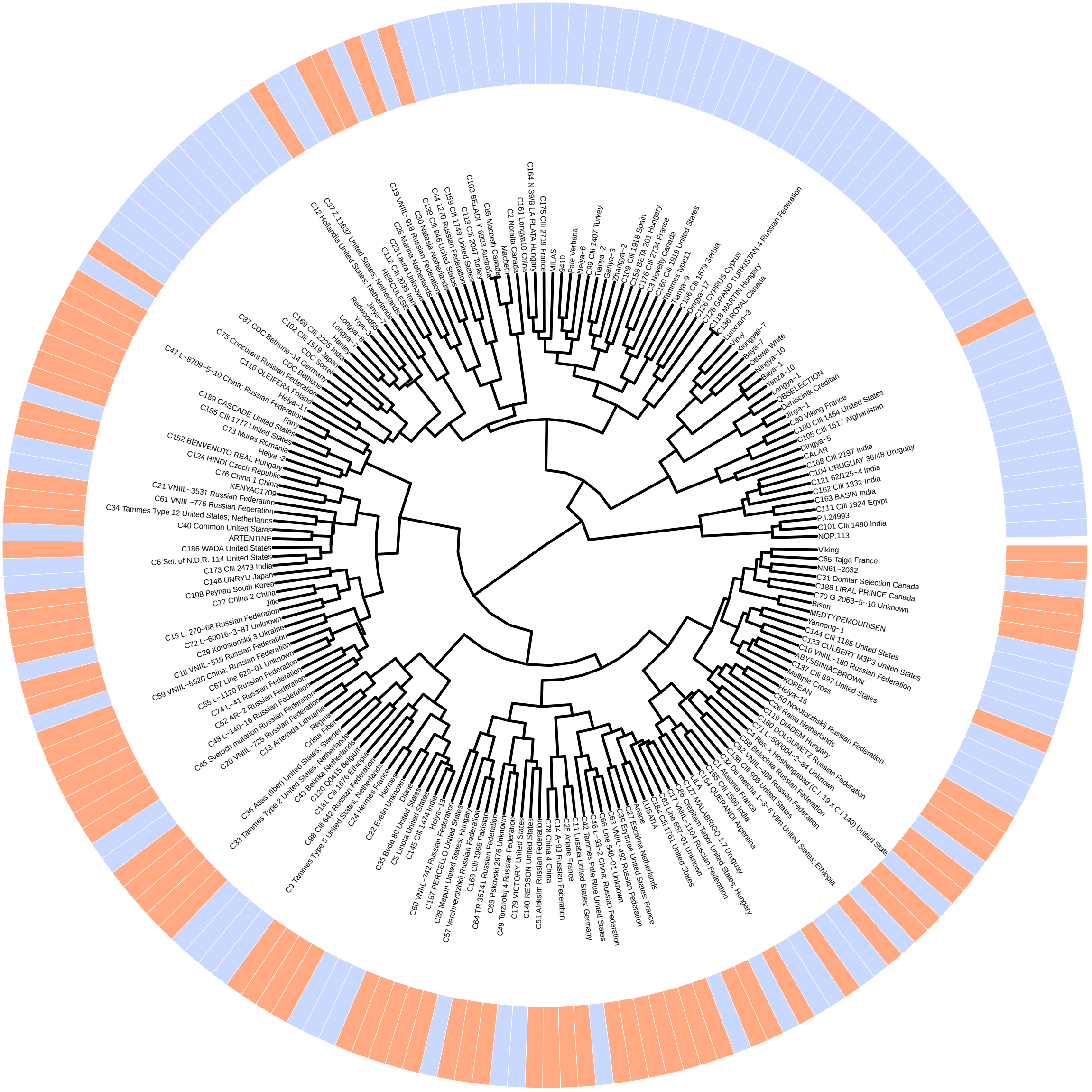

Supplementary Figure S1d. Dendrogram of linseed and fiber flax varieties based on polymorphisms of *ABC* and *HMA* genes.

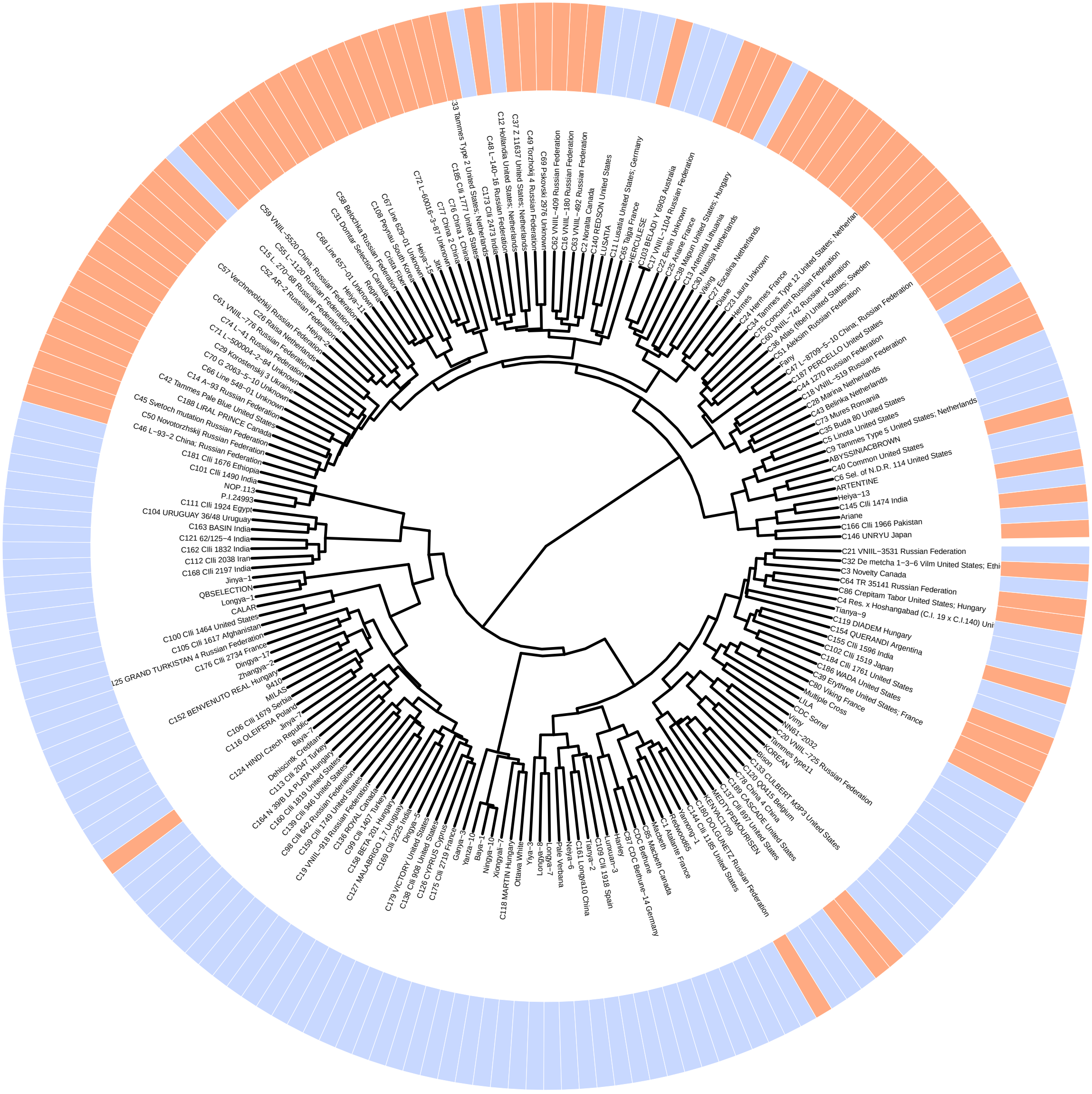

Supplementary Figure S1e. Dendrogram of linseed and fiber flax varieties based on polymorphisms of genes involved in lignan synthesis (*DIR*, *PLR*, and *UGT* families).

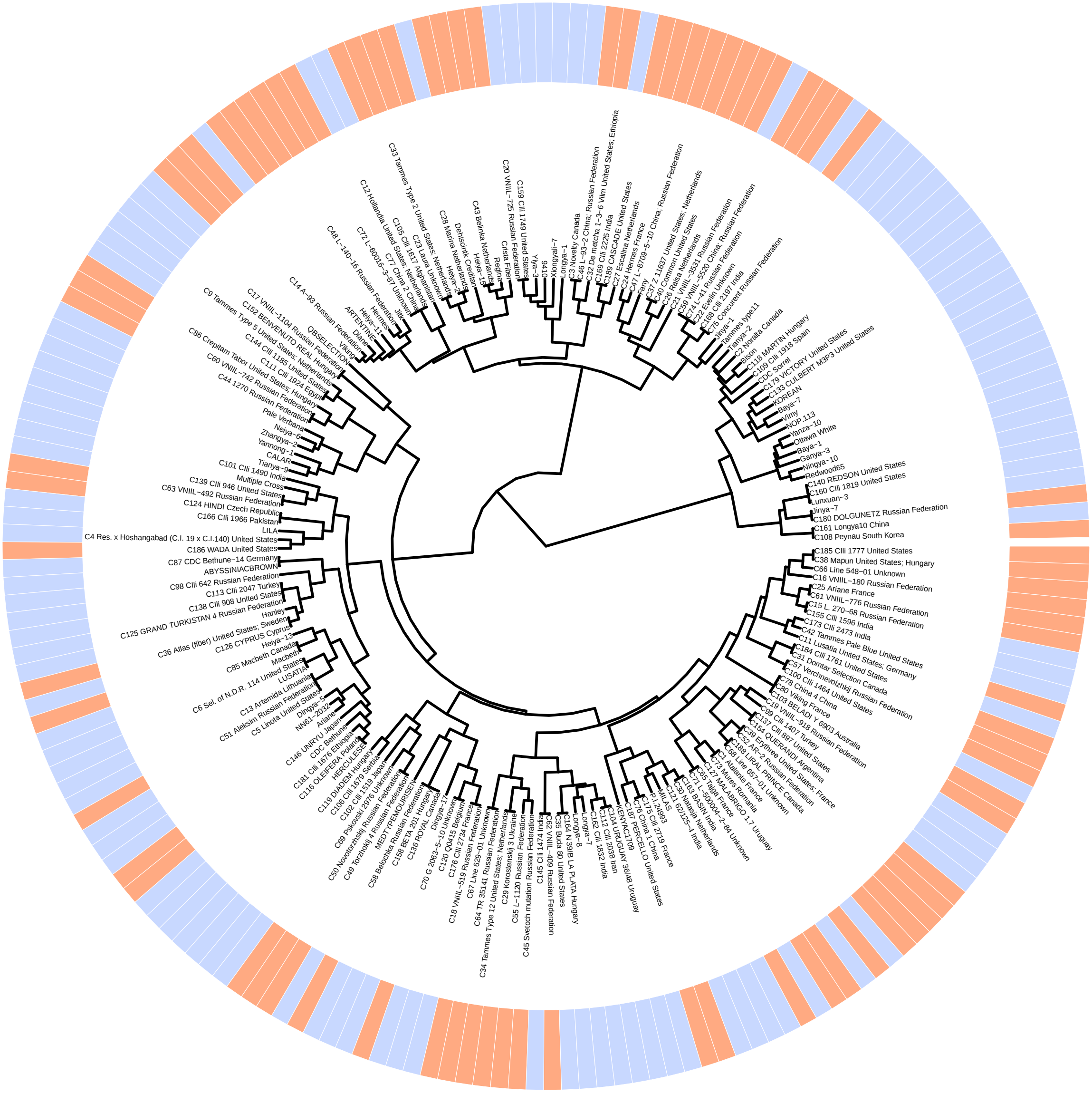

Supplementary Figure S1f. Dendrogram of linseed and fiber flax varieties based on polymorphisms of *TUB* genes.

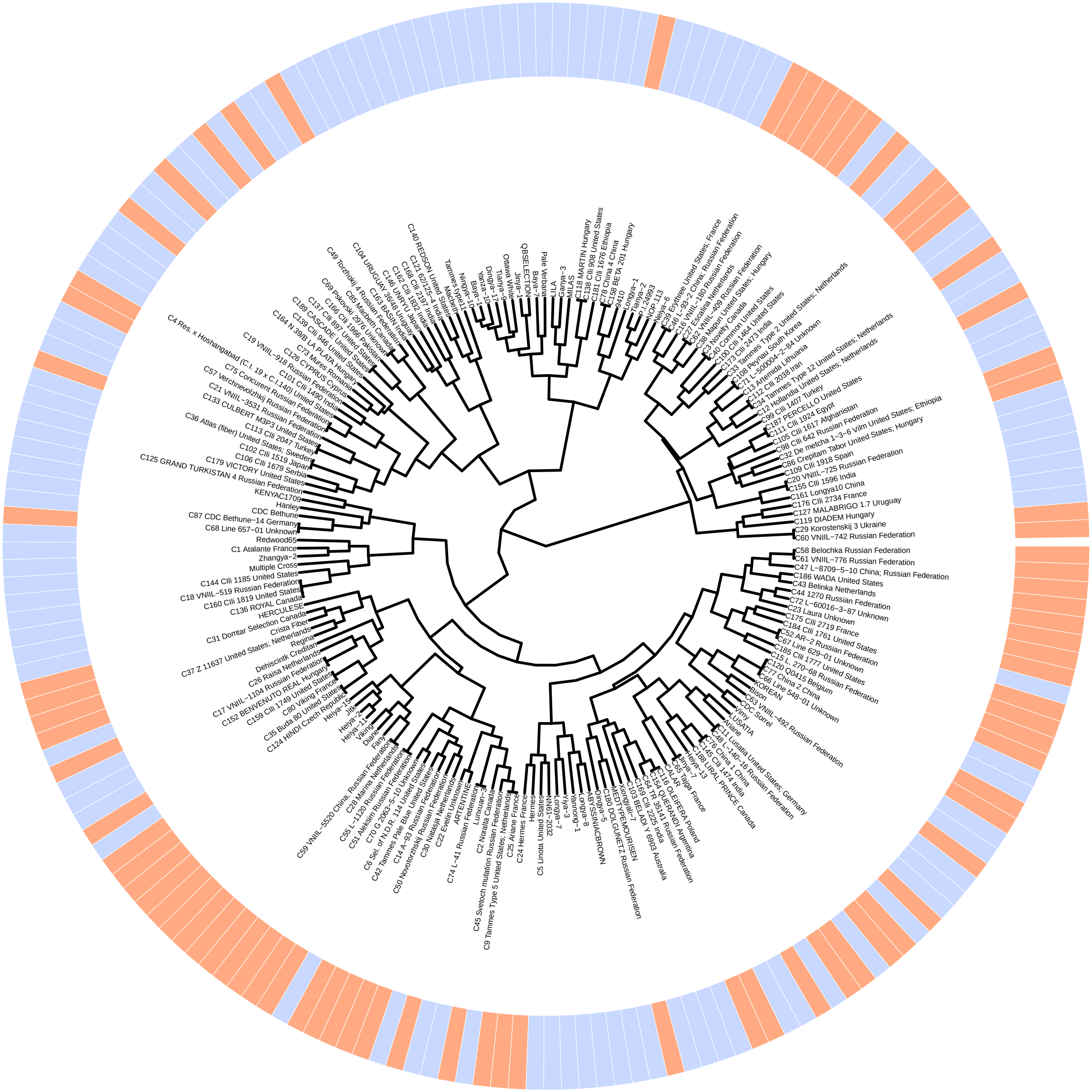

Supplementary Figure S1g. Dendrogram of linseed and fiber flax varieties based on polymorphisms of *CESA* genes.

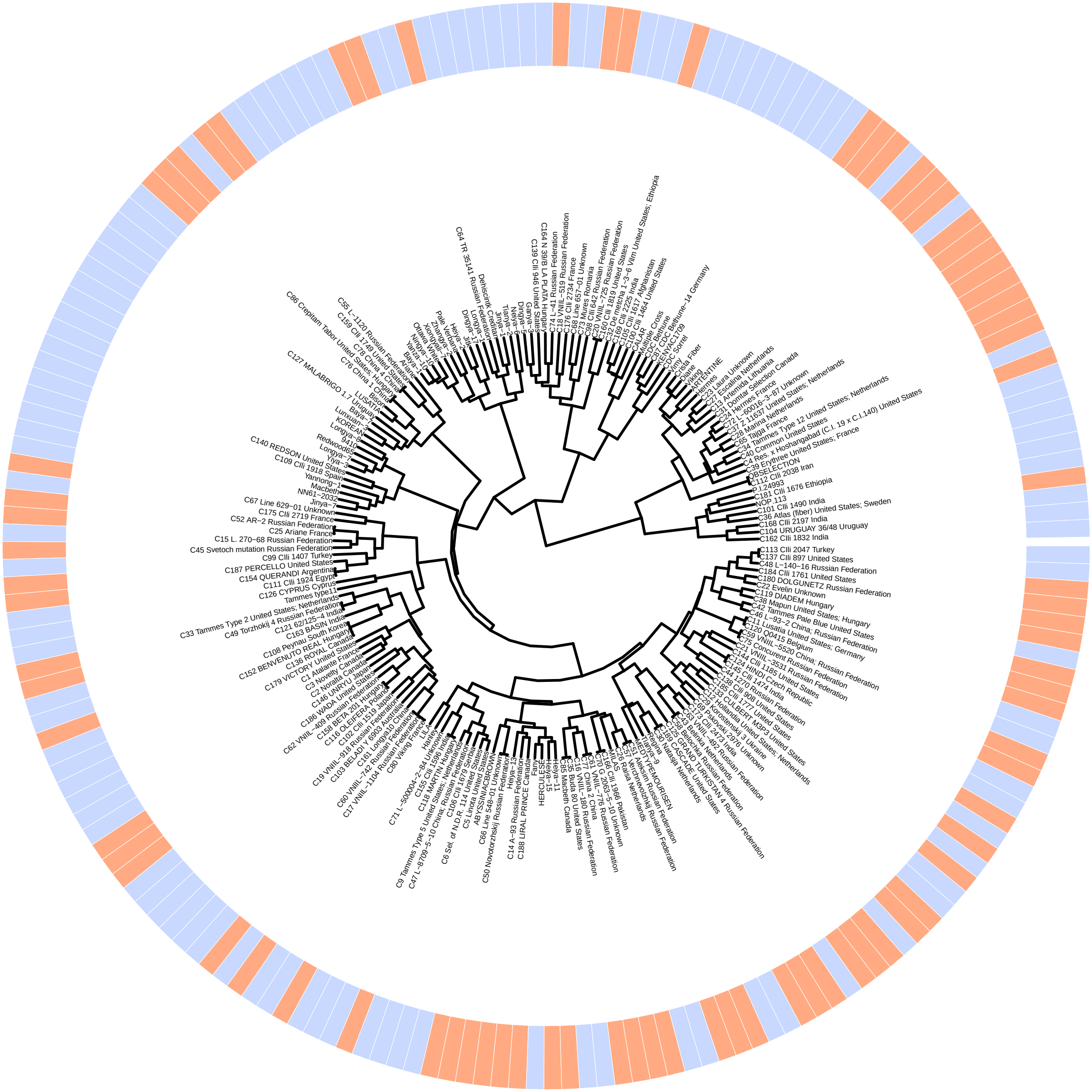

Supplementary Figure S1h. Dendrogram of linseed and fiber flax varieties based on polymorphisms of *RGL* genes.

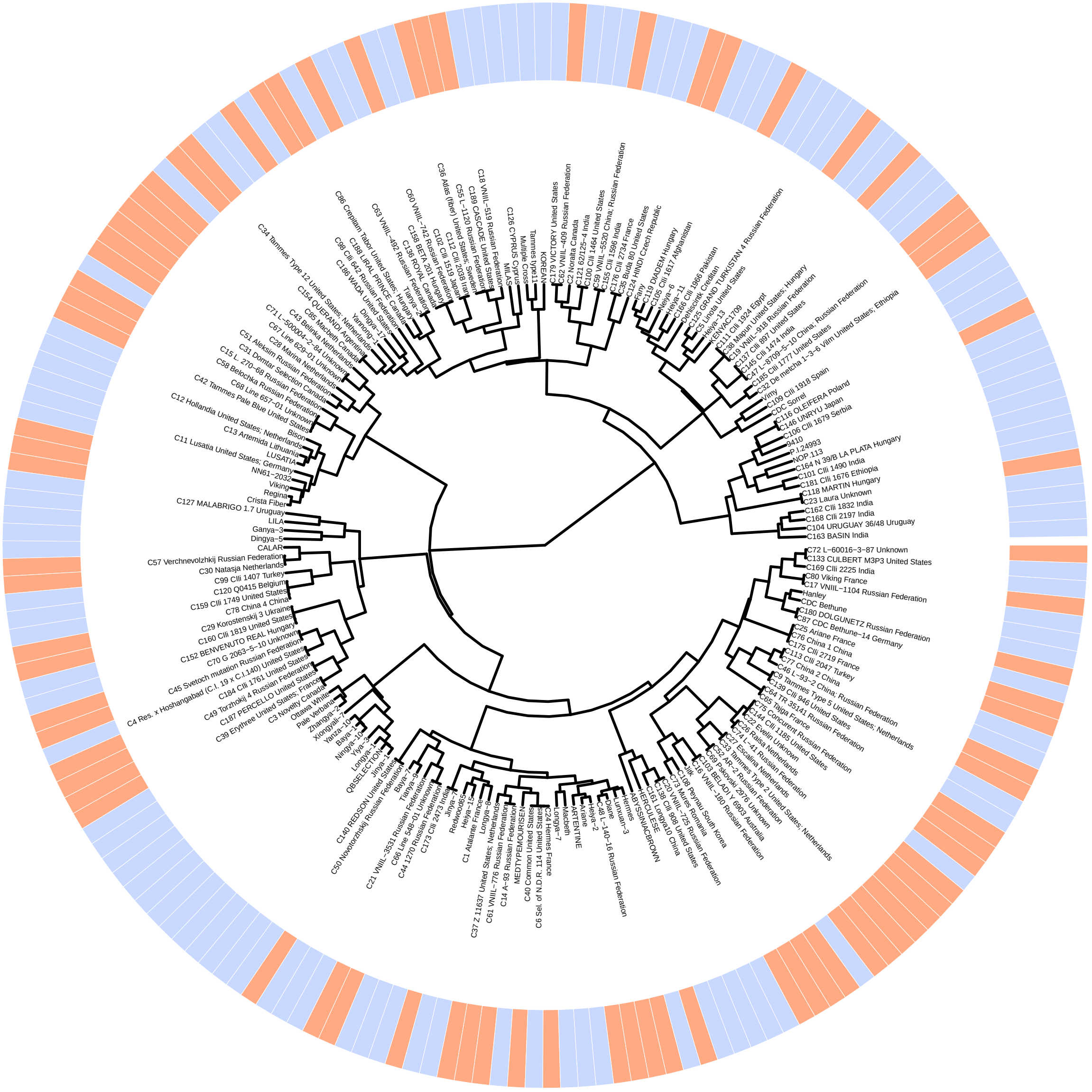

Supplementary Figure S1i. Dendrogram of linseed and fiber flax varieties based on polymorphisms of genes involved in fatty acid synthesis (*FAD* and *SAD* families).

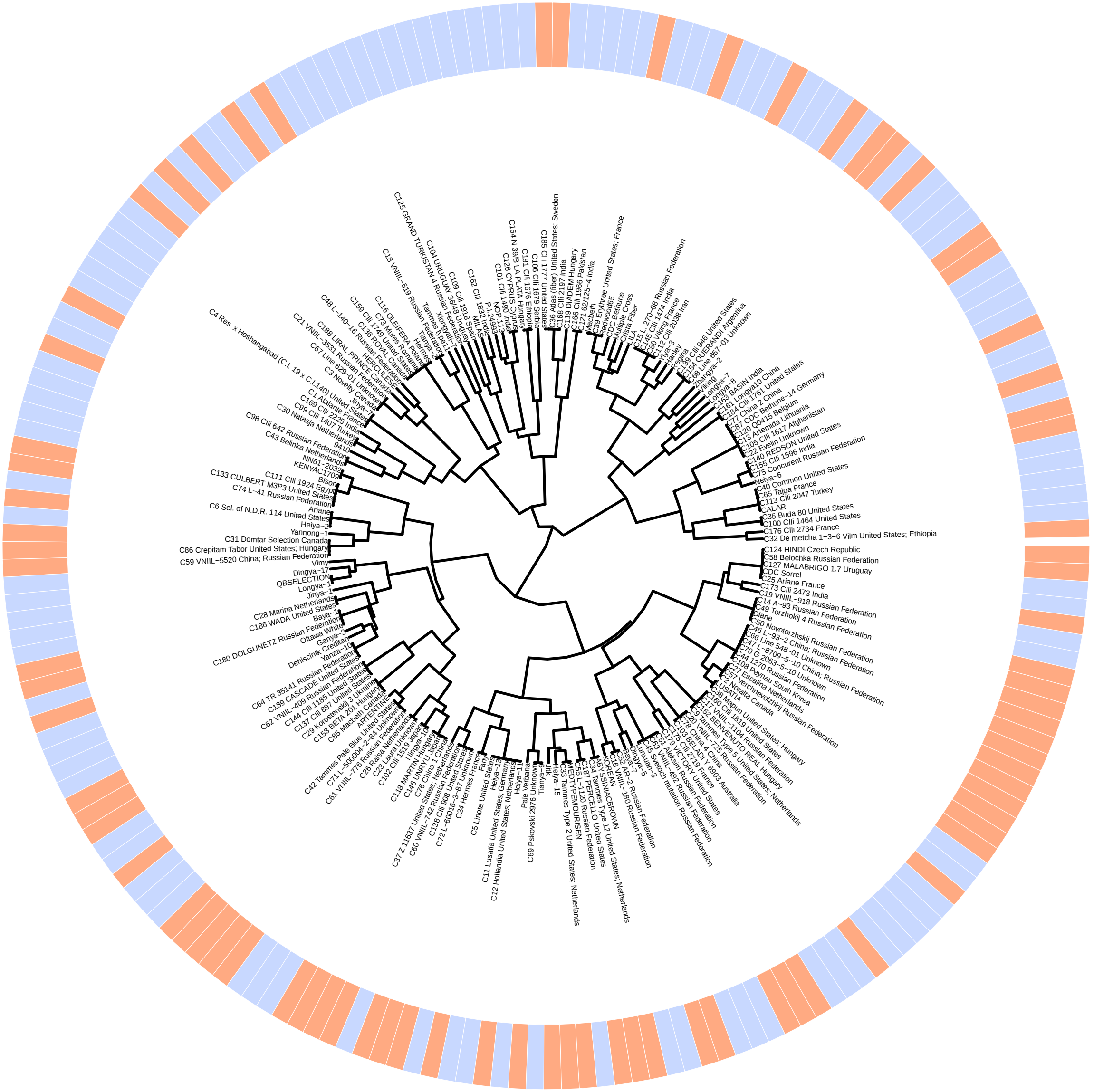

Supplementary Figure S1j. Dendrogram of linseed and fiber flax varieties based on polymorphisms of *ACT* genes.

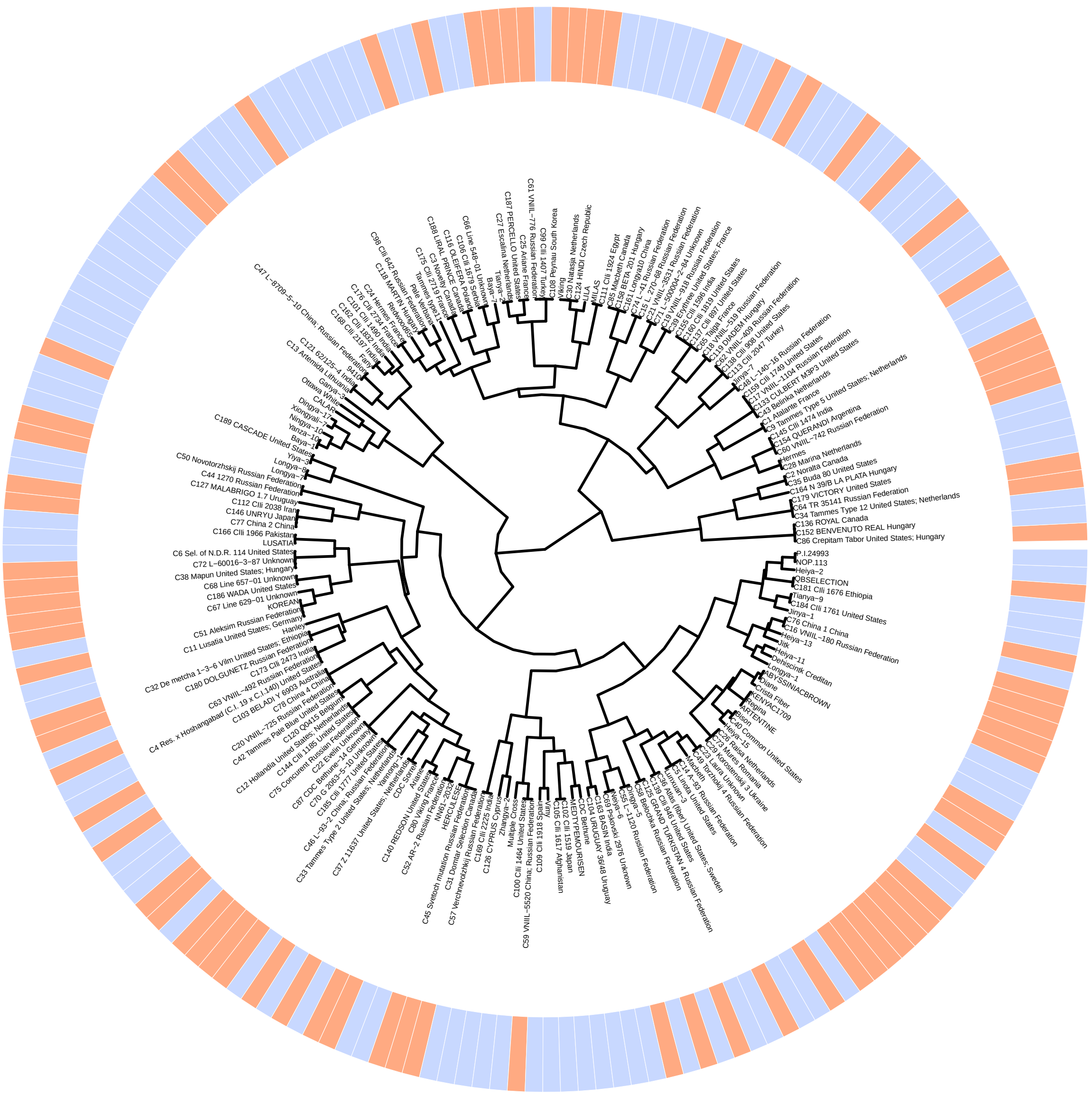

Supplement: Supplementary file 1 [file plants-10-02616-s001.zip › Supplementary Figure S1.pdf]
