# Supplementary material for: Genes Associated with the Flax Plant Type (Oil or Fiber) Identified Based on Genome and Transcriptome Sequencing Data
Source: Plants (Basel). 2021 Nov 28;10(12):2616. doi: 10.3390/plants10122616 (PMC8707629; doi:10.3390/plants10122616)
Supplement: Supplementary file 1 [file plants-10-02616-s001.zip › Supplementary Figure S3.pdf]

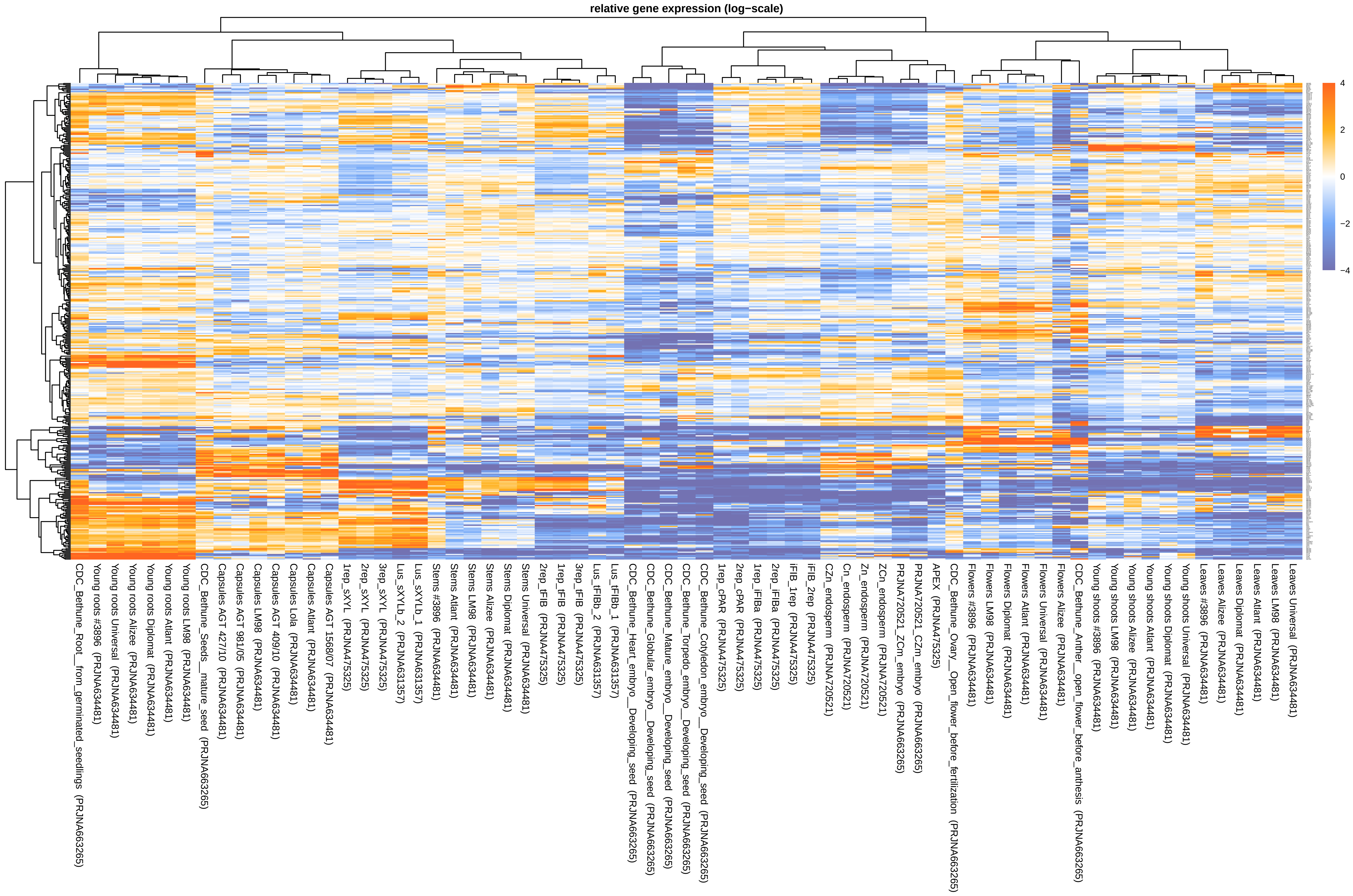

Supplementary Figure S3. Heatmap of expression levels of genes of *4CL*, *C3'H*, *C4H*, *CAD*, *CCR*, *CCoAOMT*, *COMT*, *F5H*, *HCT*, *PAL*, *CTL*, *BGAL*, *ABC*, *HMA*, *DIR*, *PLR*, *UGT*, *TUB*, *CESA*, *RGL*, *FAD*, *SAD*, and *ACT* families in different flax tissues (individual samples).
