# Supplementary material for: Genes Associated with the Flax Plant Type (Oil or Fiber) Identified Based on Genome and Transcriptome Sequencing Data
Source: Plants (Basel). 2021 Nov 28;10(12):2616. doi: 10.3390/plants10122616 (PMC8707629; doi:10.3390/plants10122616)

Supplementary Figure S4a. Dendrogram of linseed and fiber flax varieties based on polymorphisms of *PAL1* gene. Linseed varieties are marked in blue and fiber flax ones are marked in red.

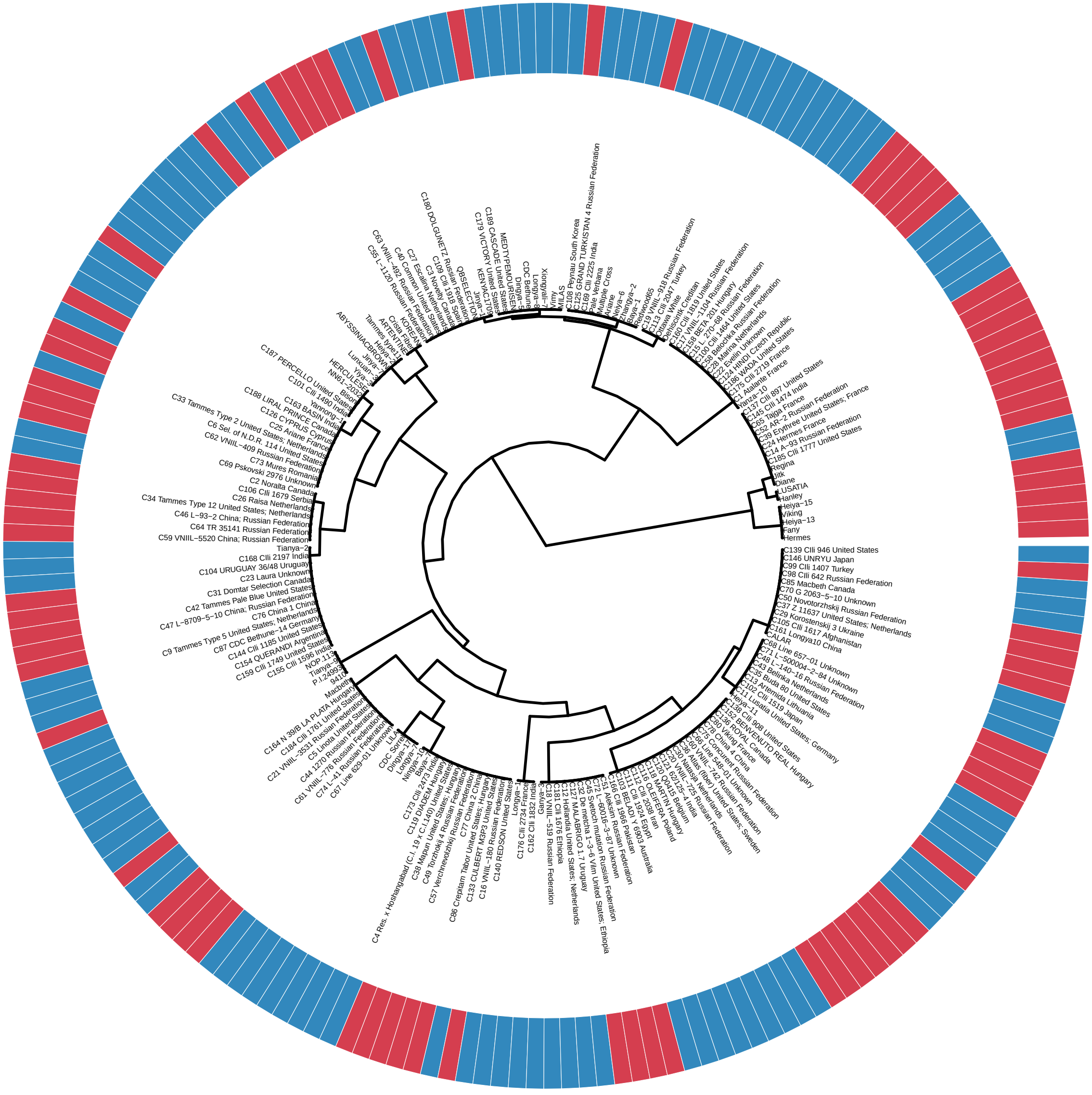

Supplementary Figure S4b. Dendrogram of linseed and fiber flax varieties based on polymorphisms of *PAL3* gene.

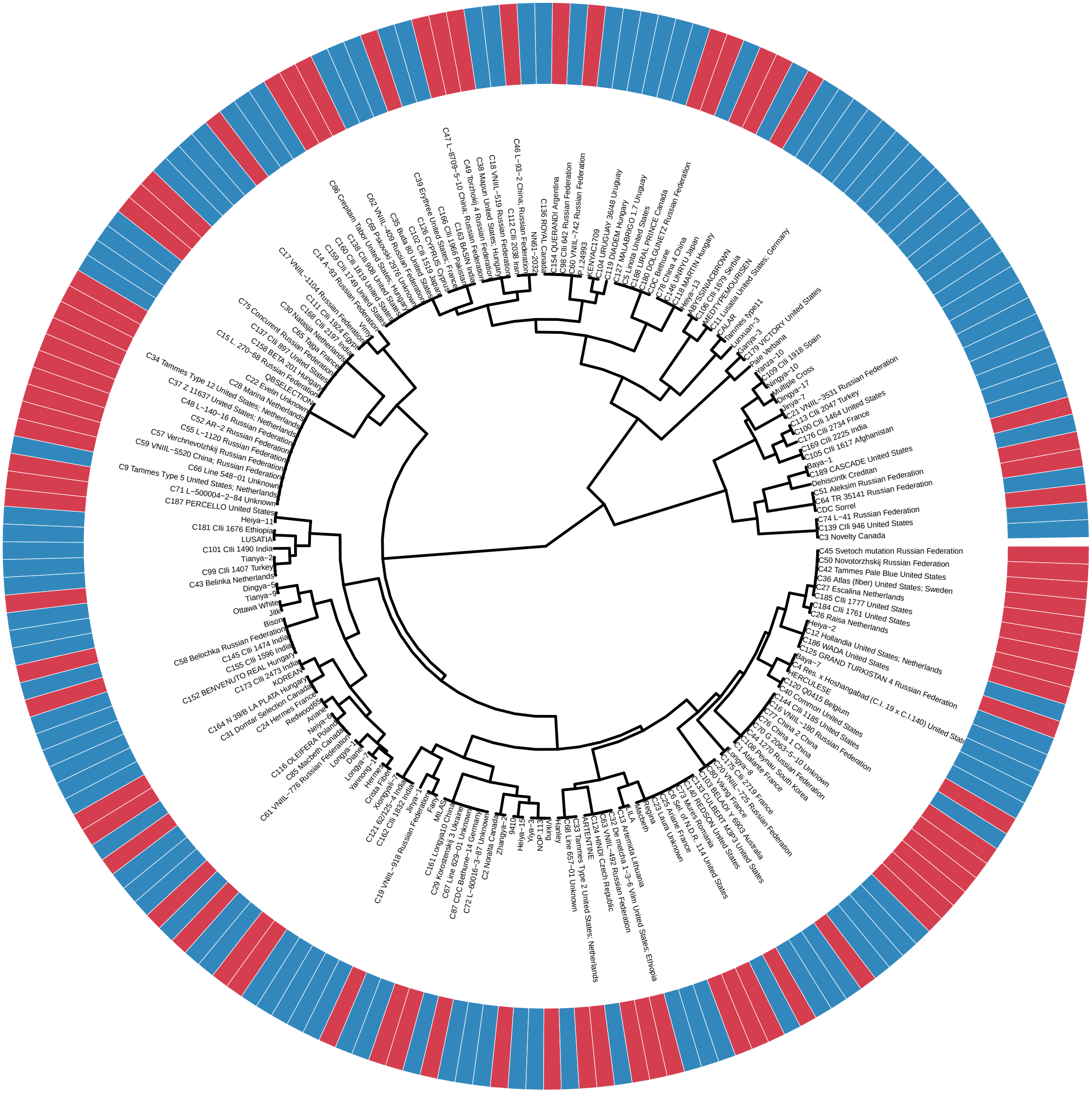

Supplementary Figure S4c. Dendrogram of linseed and fiber flax varieties based on polymorphisms of *4CL1* gene.

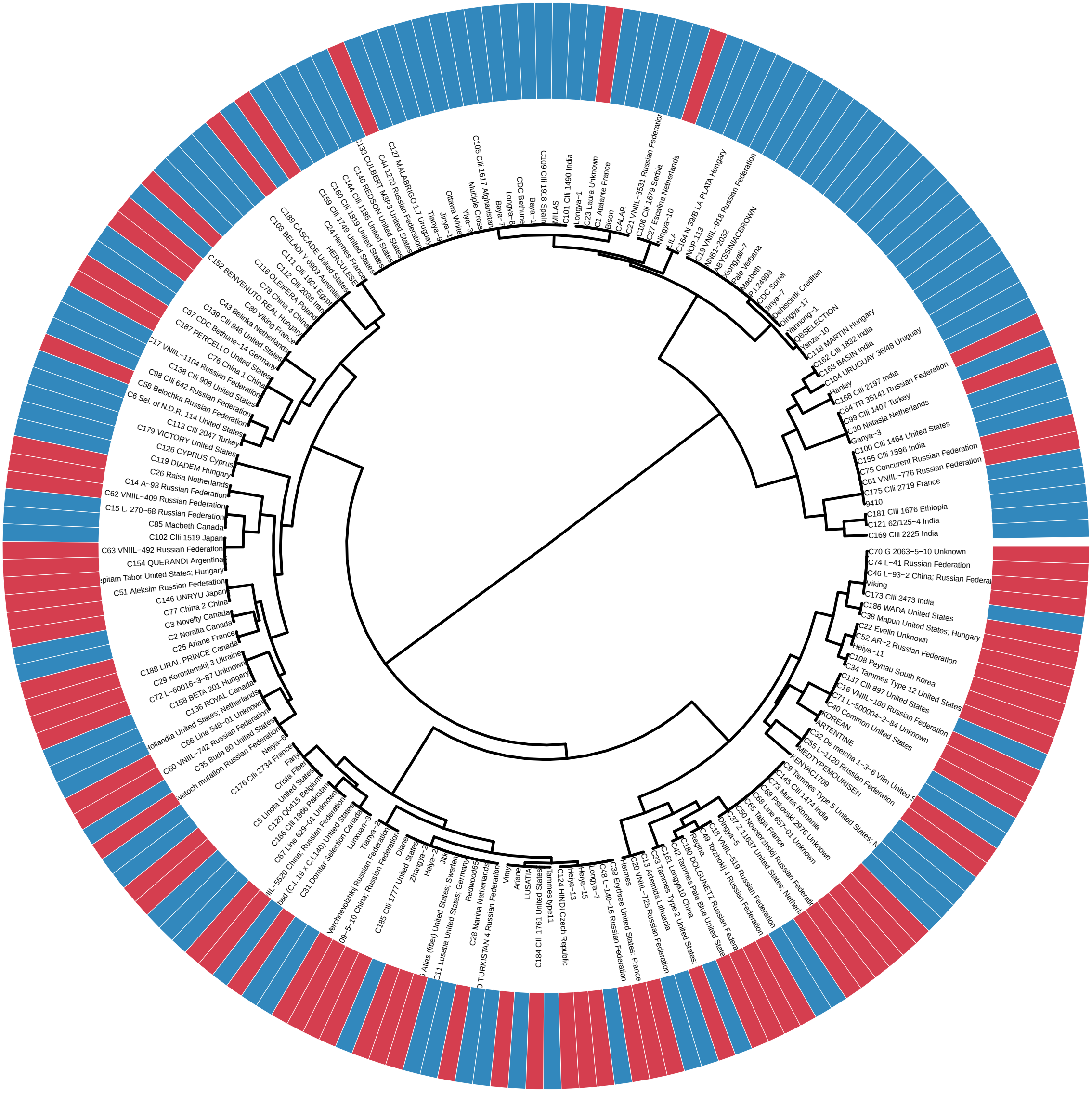

Supplementary Figure S4d. Dendrogram of linseed and fiber flax varieties based on polymorphisms of *C4H4* gene.

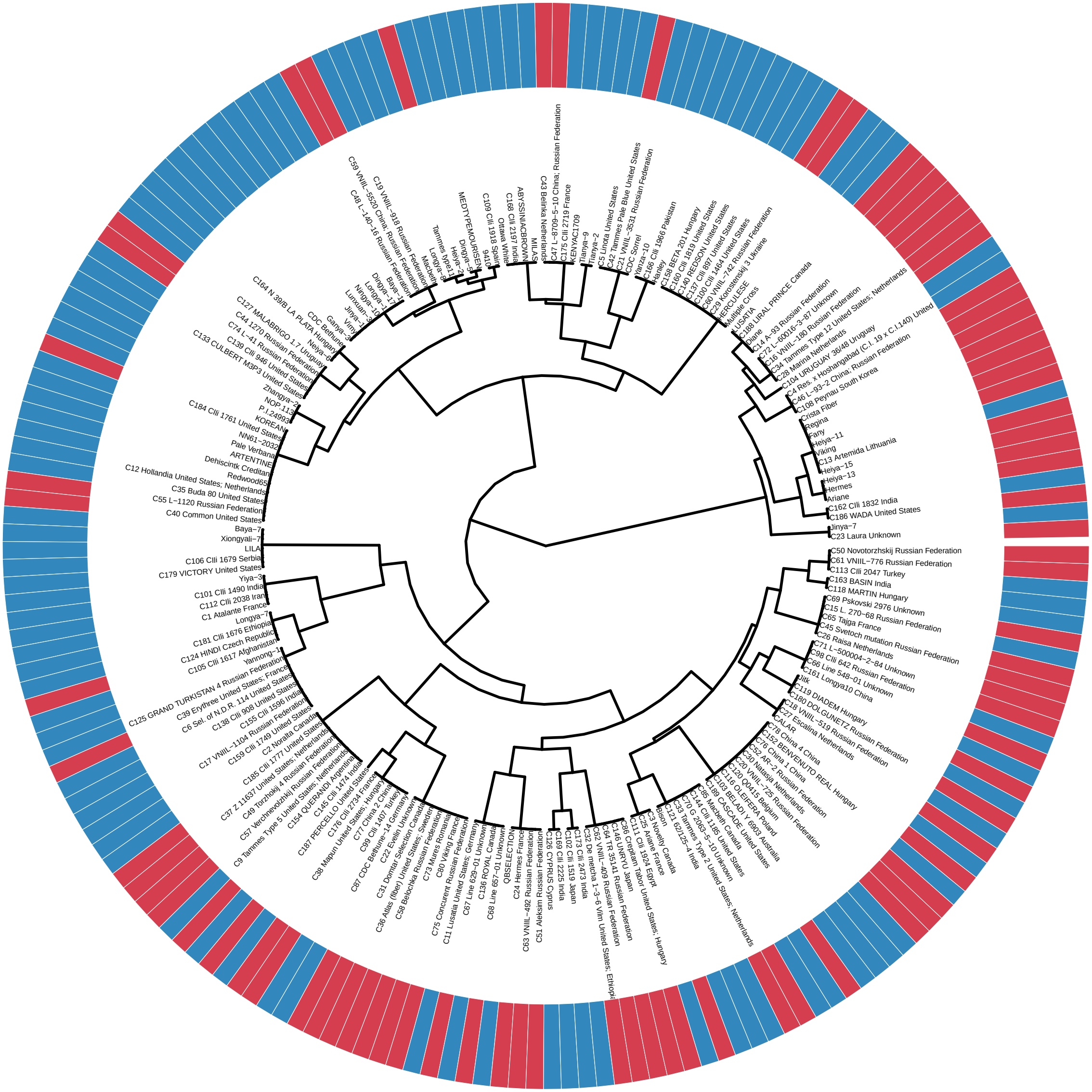

Supplementary Figure S4e. Dendrogram of linseed and fiber flax varieties based on polymorphisms of *CAD1B* gene.

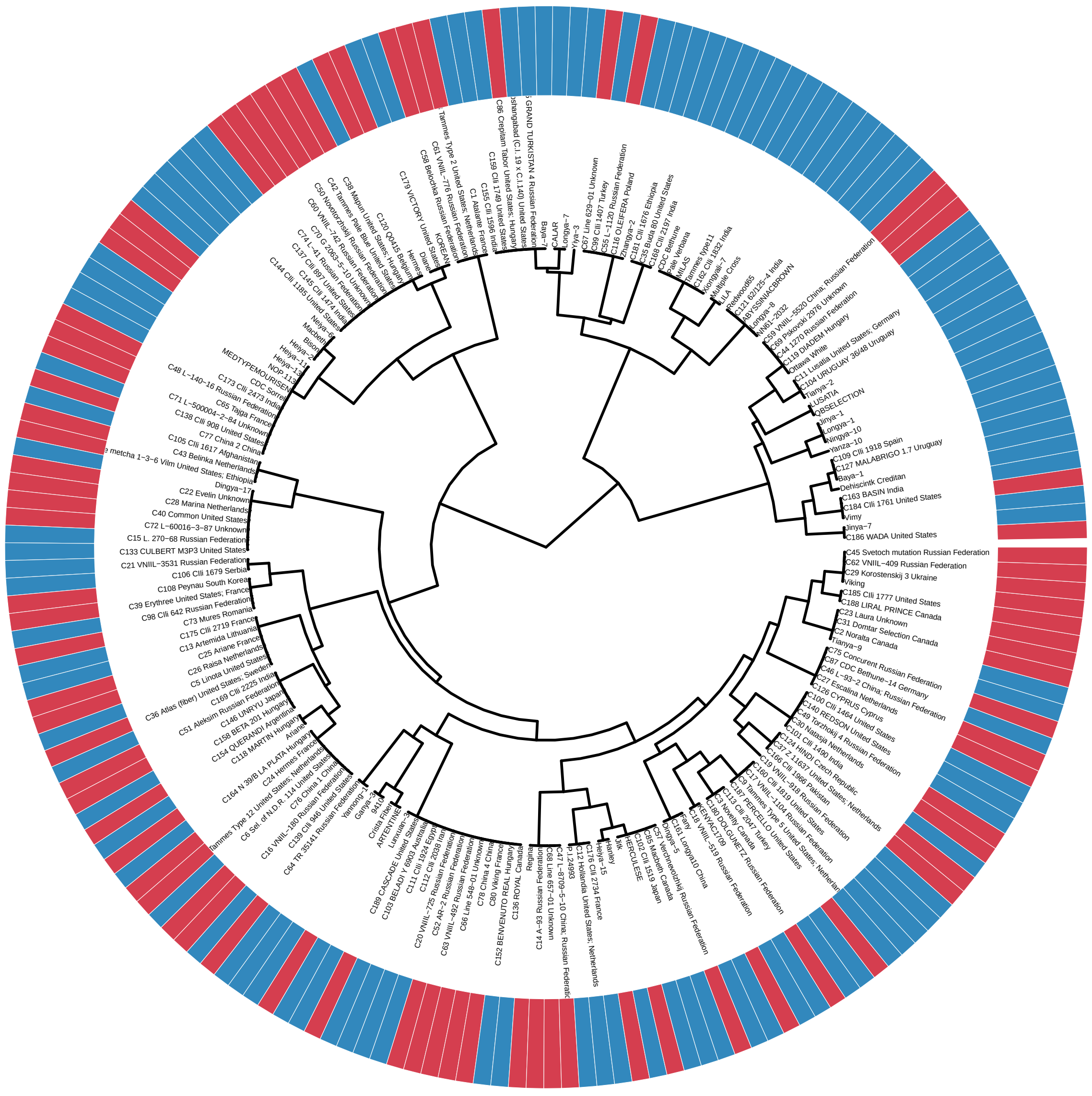

Supplementary Figure S4f. Dendrogram of linseed and fiber flax varieties based on polymorphisms of *CESA1-B* gene.

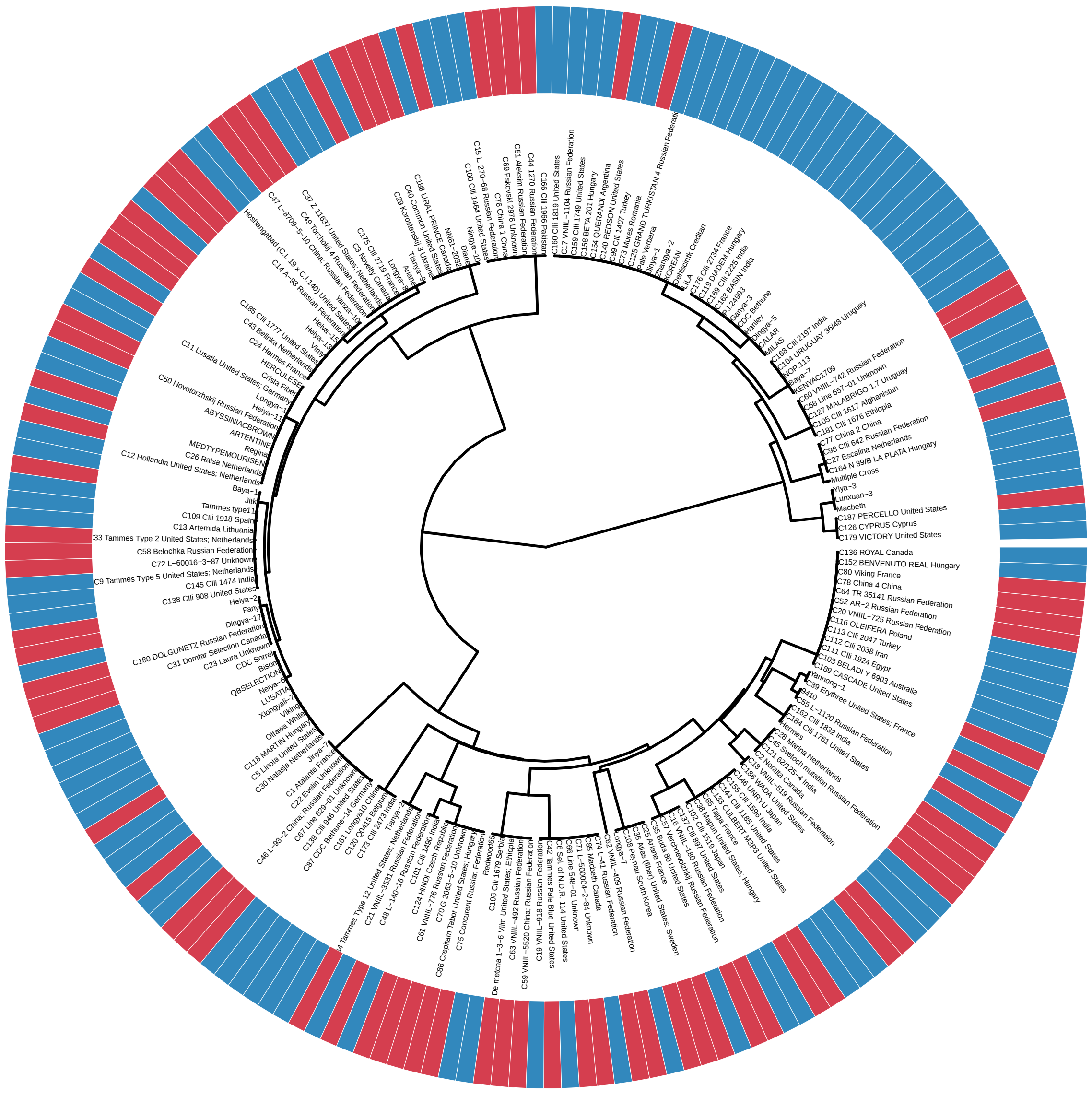

Supplementary Figure S4g. Dendrogram of linseed and fiber flax varieties based on polymorphisms of *CTL1* gene.

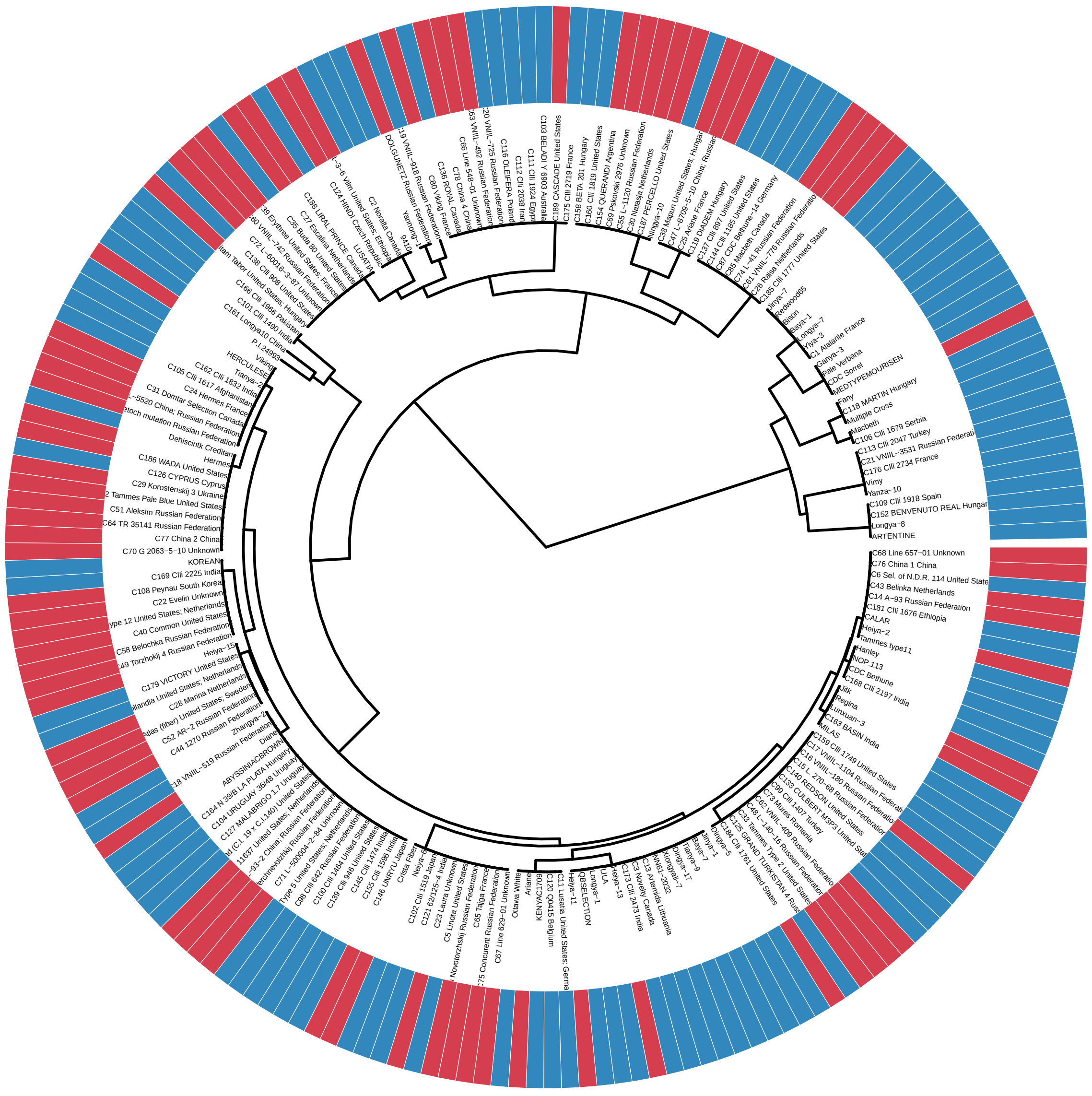

Supplementary Figure S4h. Dendrogram of linseed and fiber flax varieties based on polymorphisms of *CTL18* gene.

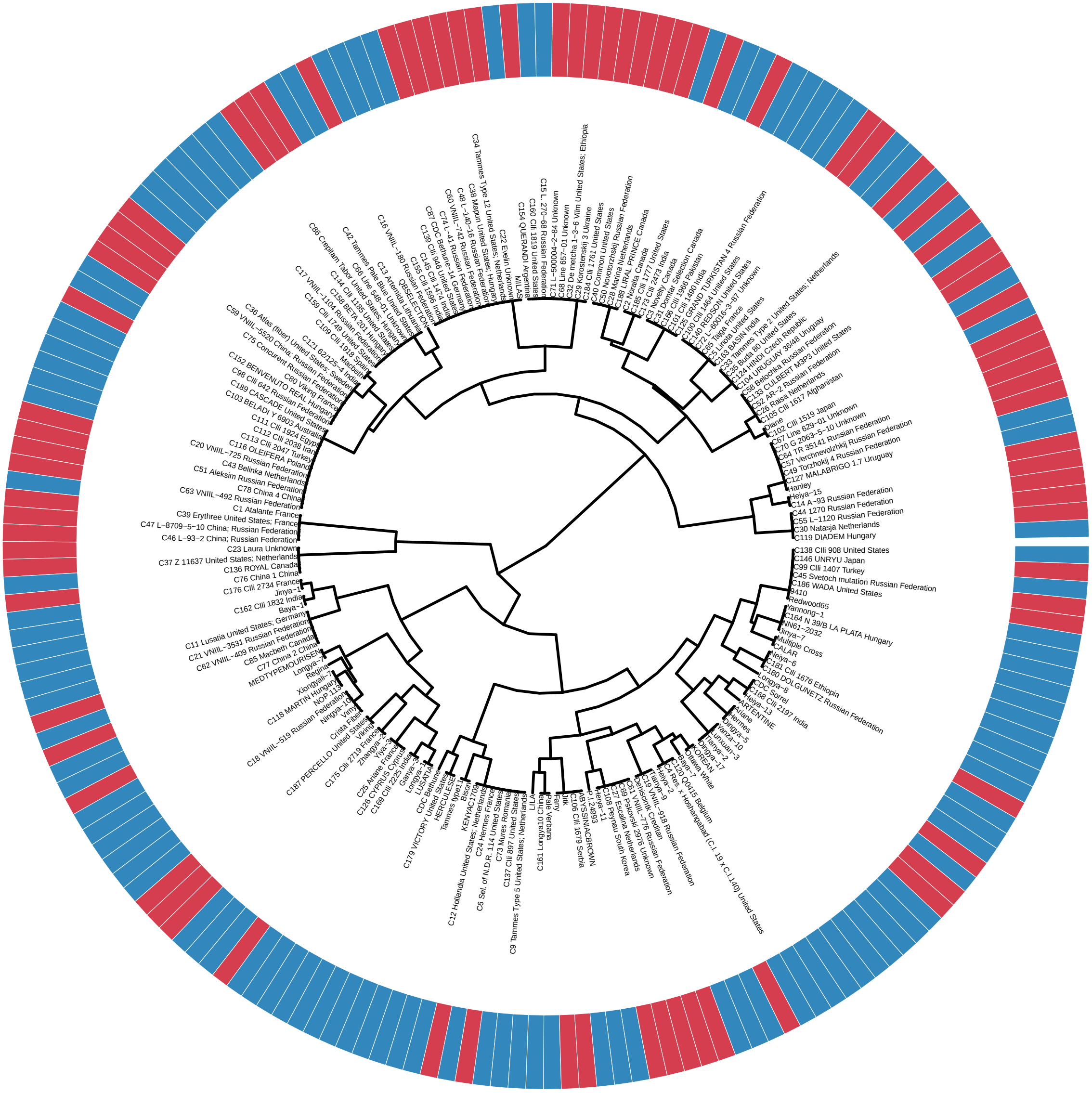

Supplementary Figure S4i. Dendrogram of linseed and fiber flax varieties based on polymorphisms of *Beta_TUB3* gene.

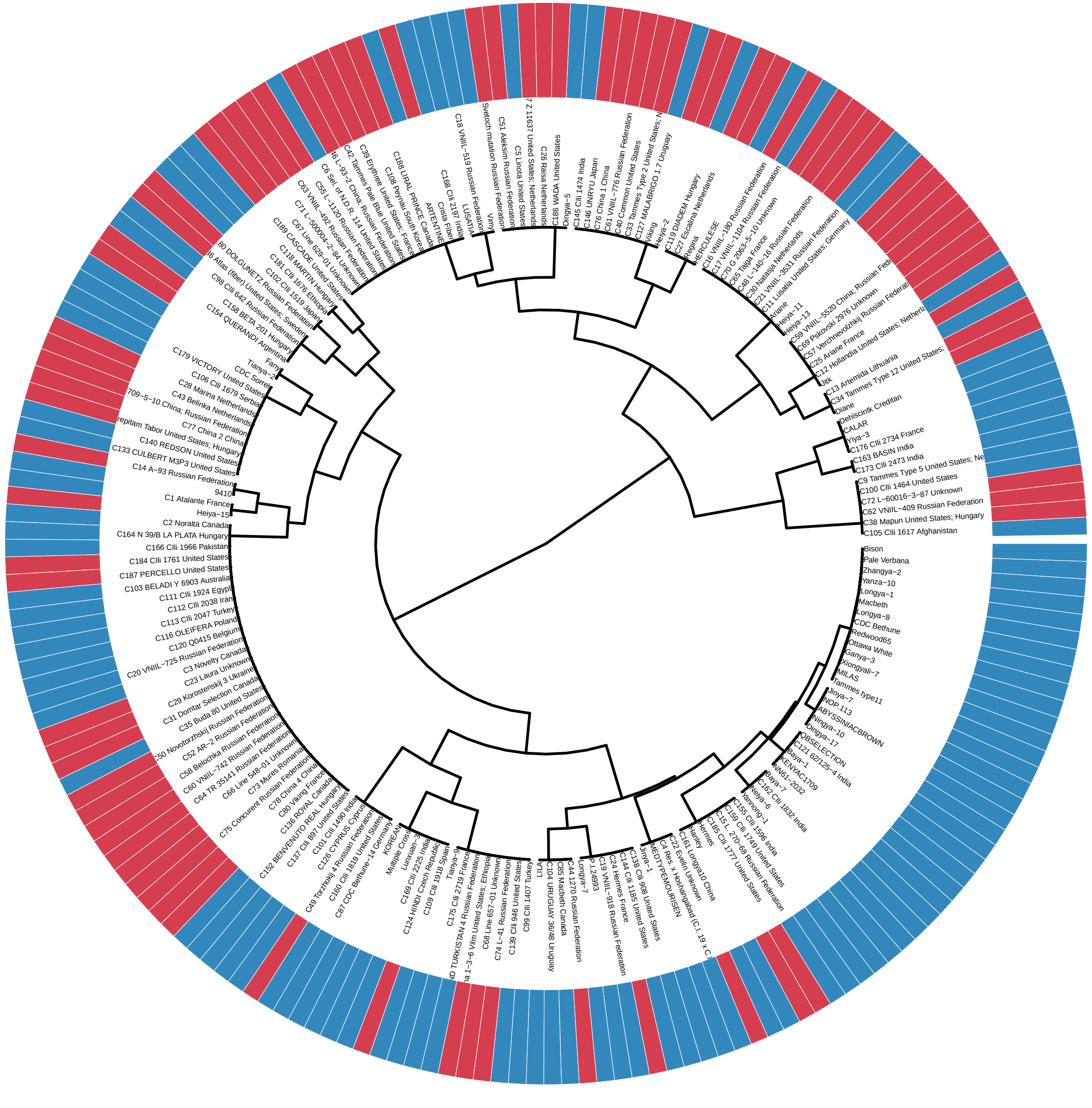

Supplementary Figure S4j. Dendrogram of linseed and fiber flax varieties based on polymorphisms of *BGAL40* gene.

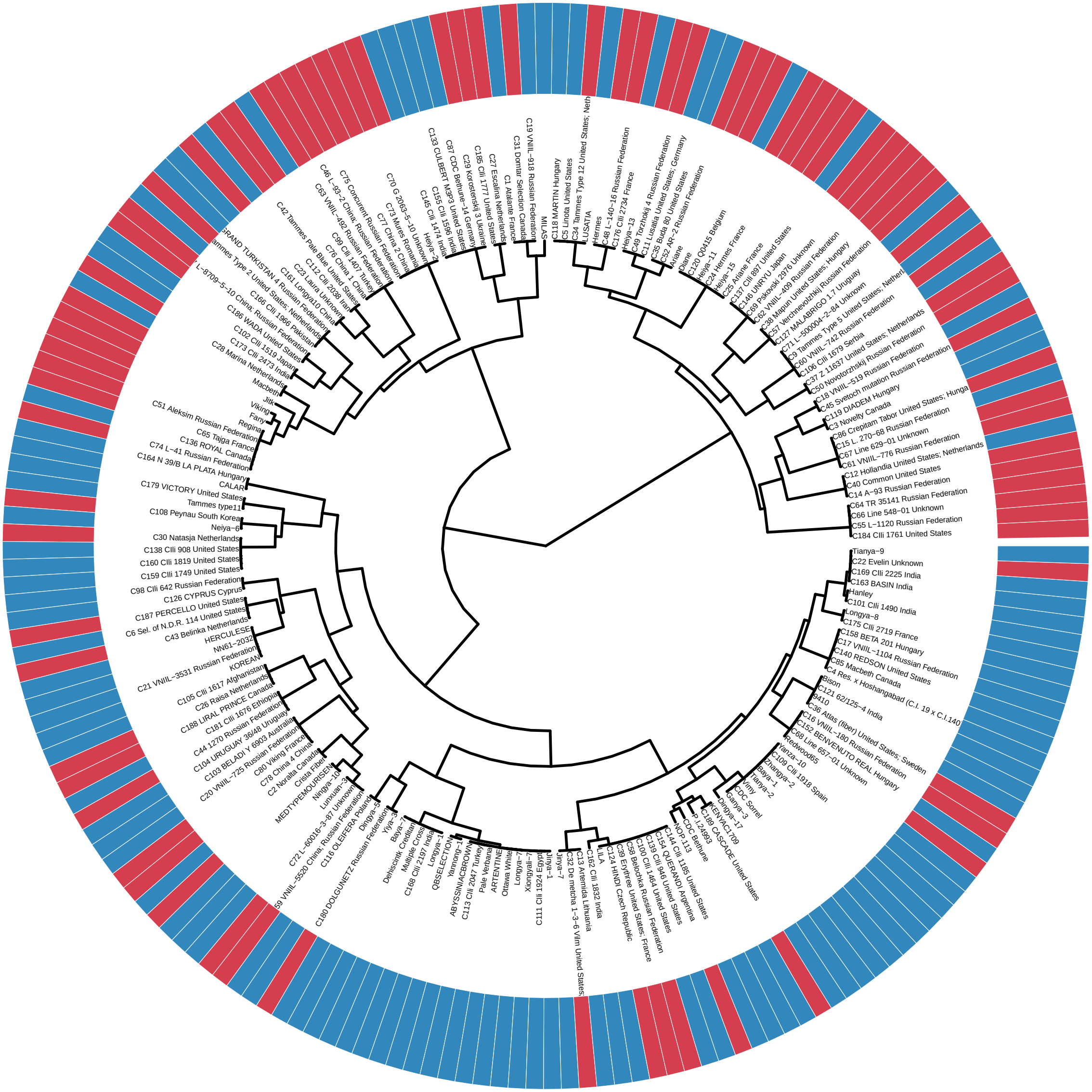

Supplementary Figure S4k. Dendrogram of linseed and fiber flax varieties based on polymorphisms of *BGAL30* gene.

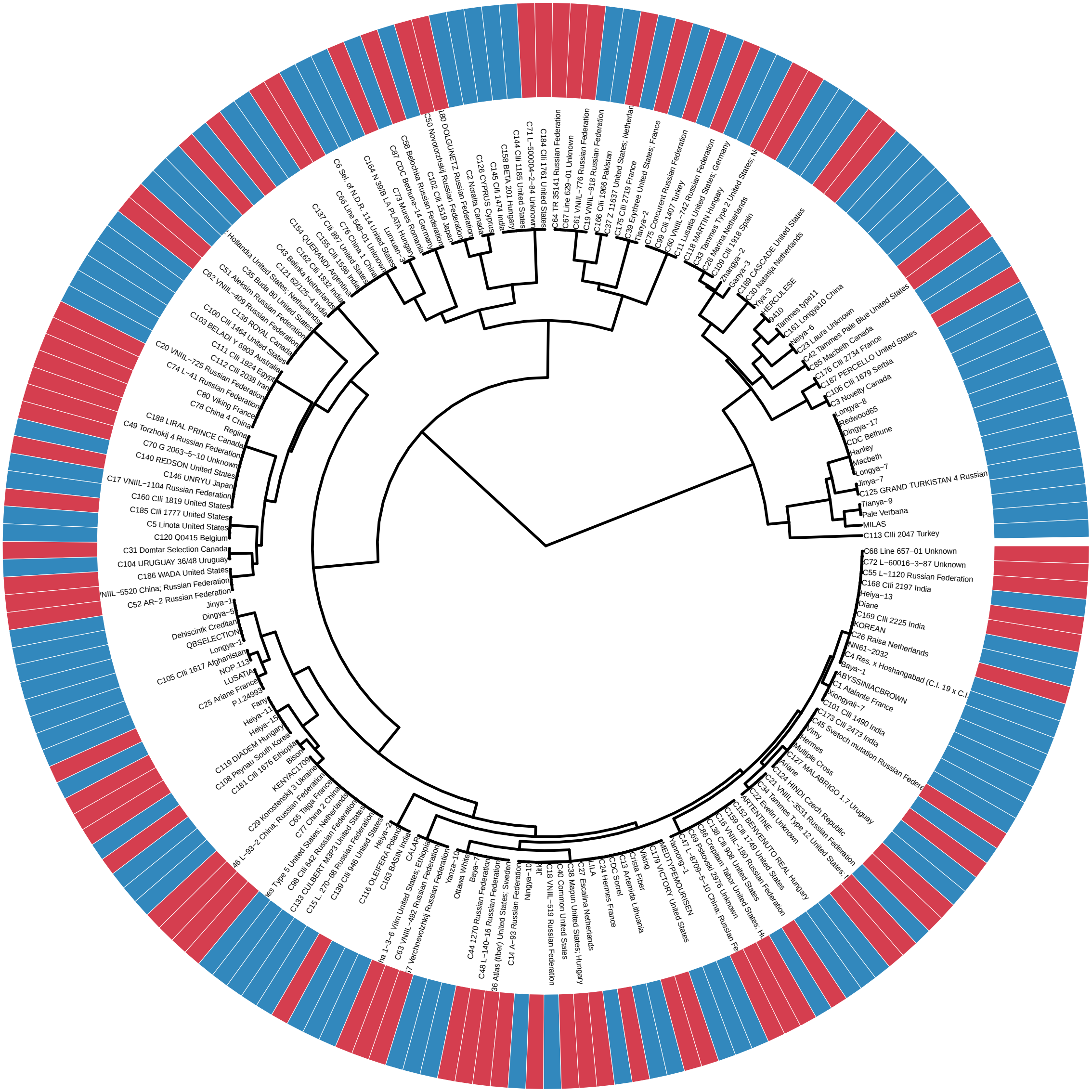

Supplementary Figure S4l. Dendrogram of linseed and fiber flax varieties based on polymorphisms of *PLR1* gene.

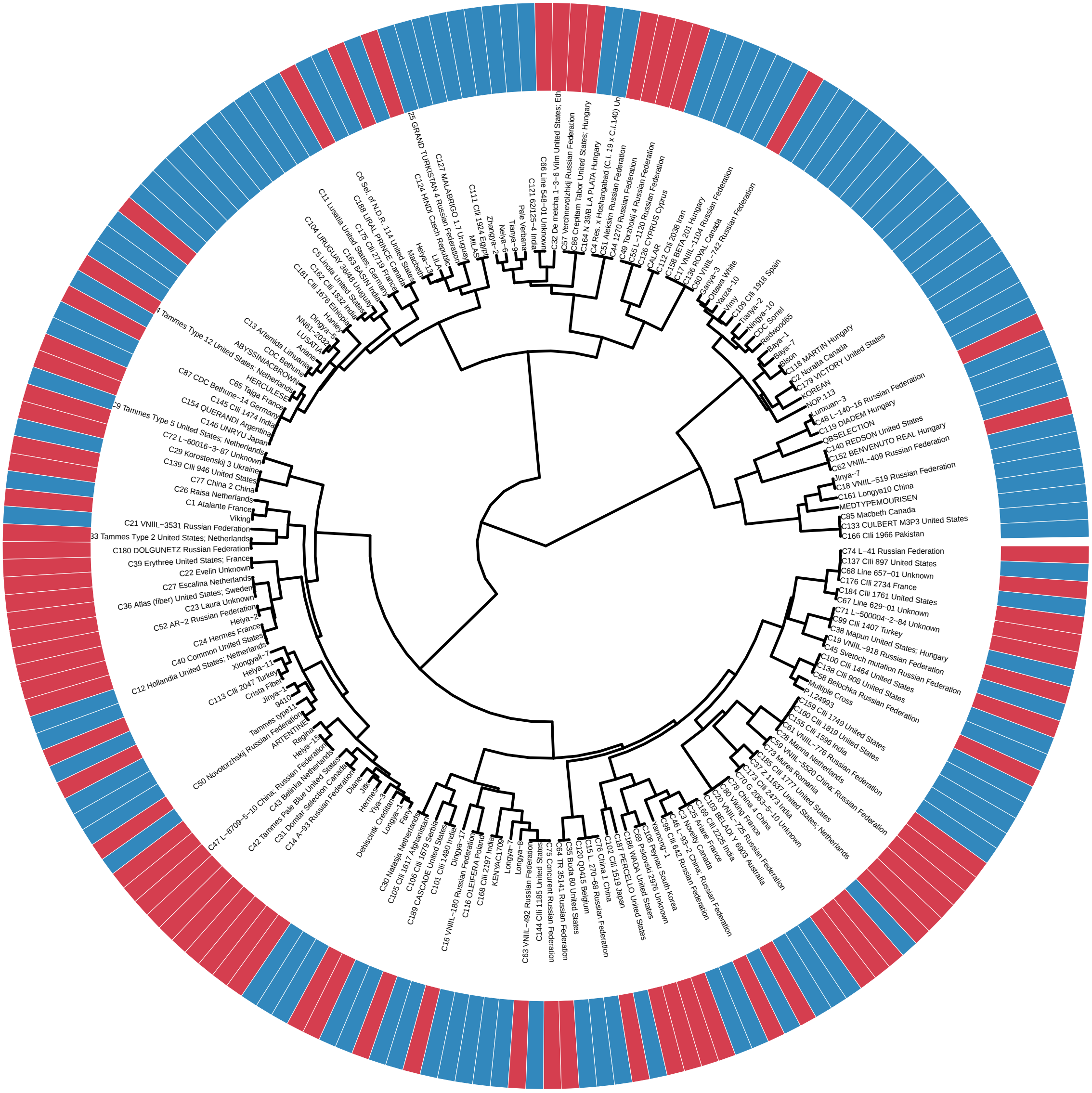

Supplementary Figure S4m. Dendrogram of linseed and fiber flax varieties based on polymorphisms of *FAD2A* gene.

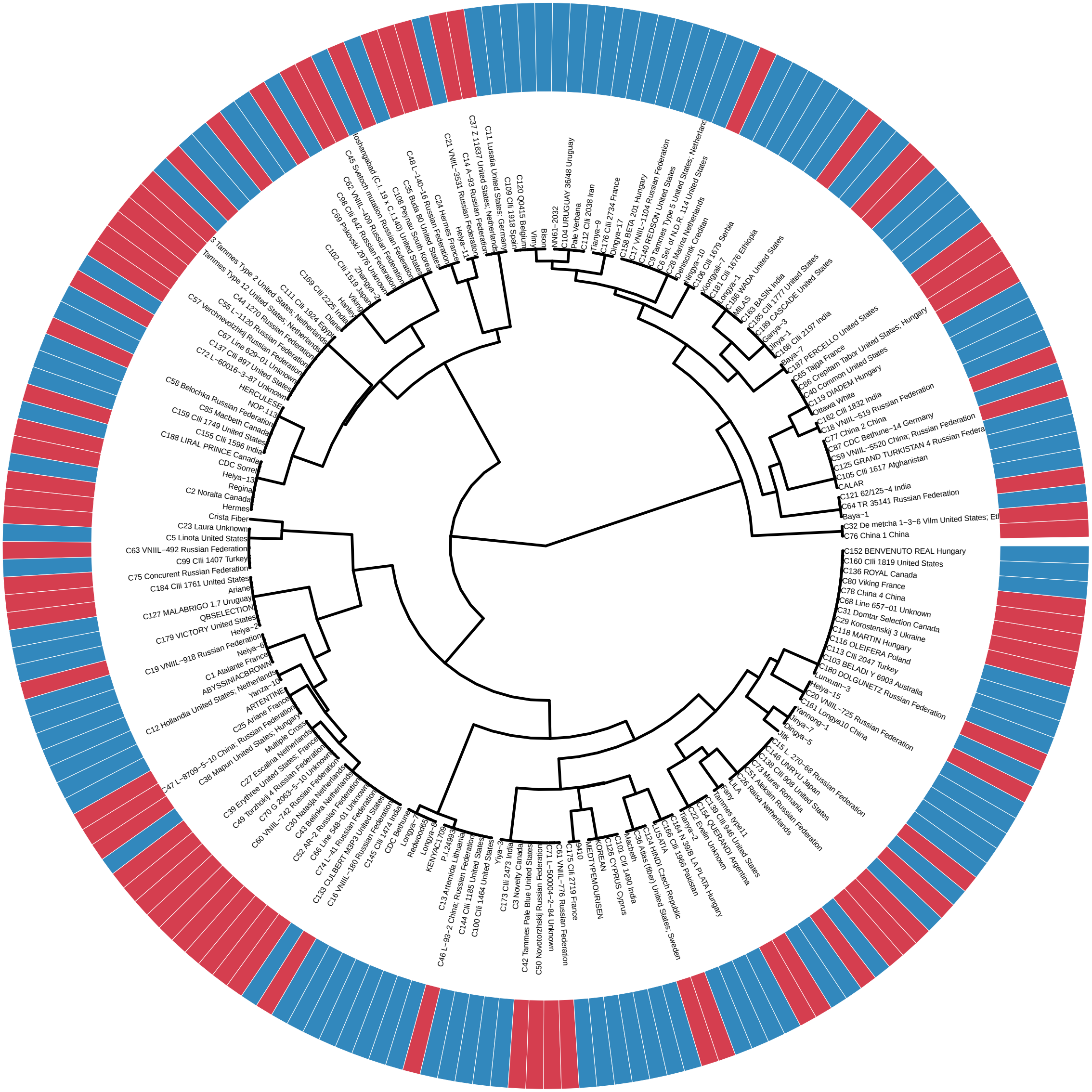

Supplementary Figure S4n. Dendrogram of linseed and fiber flax varieties based on polymorphisms of *ABCA1* gene.

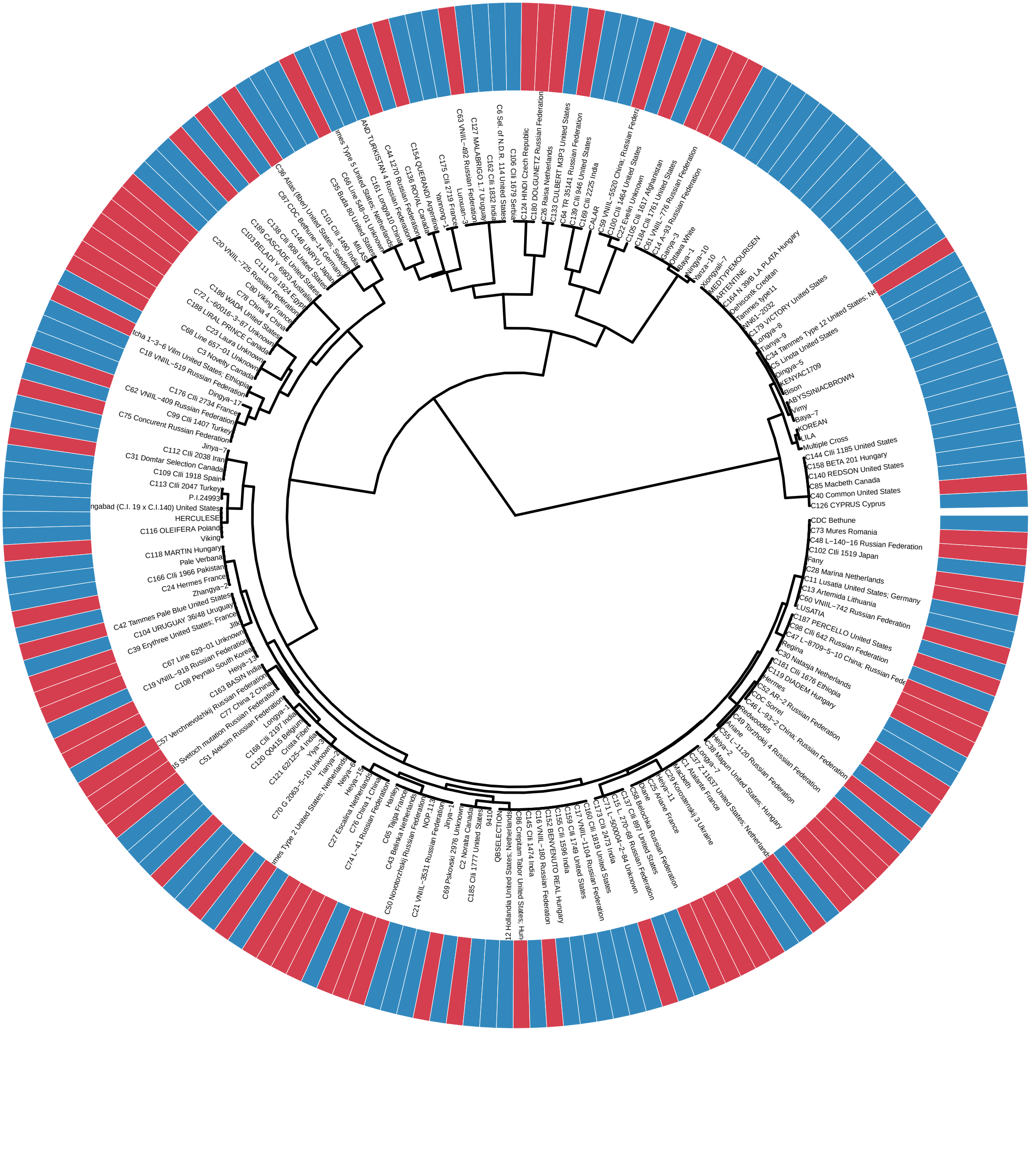

Supplementary Figure S4o. Dendrogram of linseed and fiber flax varieties based on polymorphisms of *ABCA7* gene.

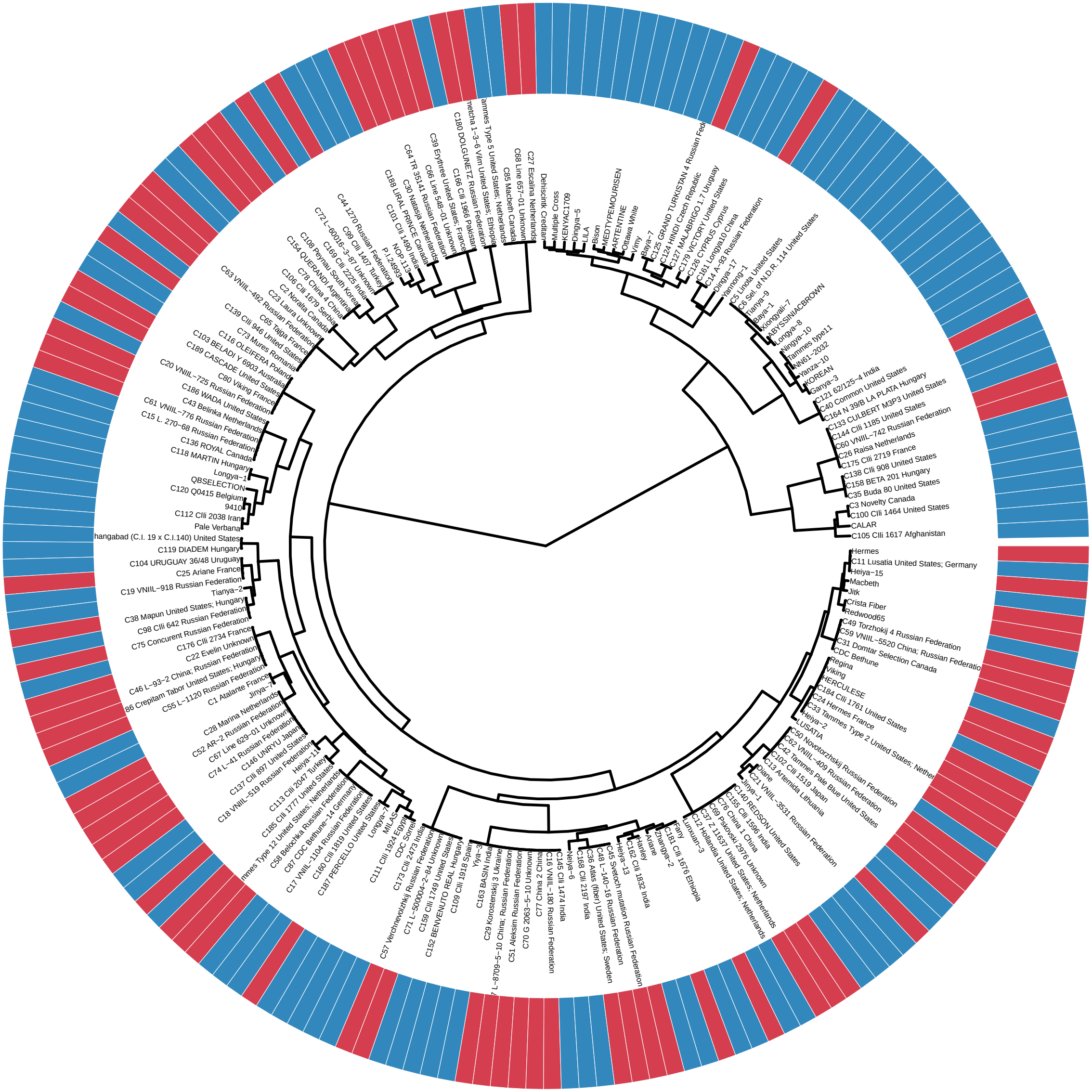

Supplementary Figure S4p. Dendrogram of linseed and fiber flax varieties based on polymorphisms of *ABCB42* gene.

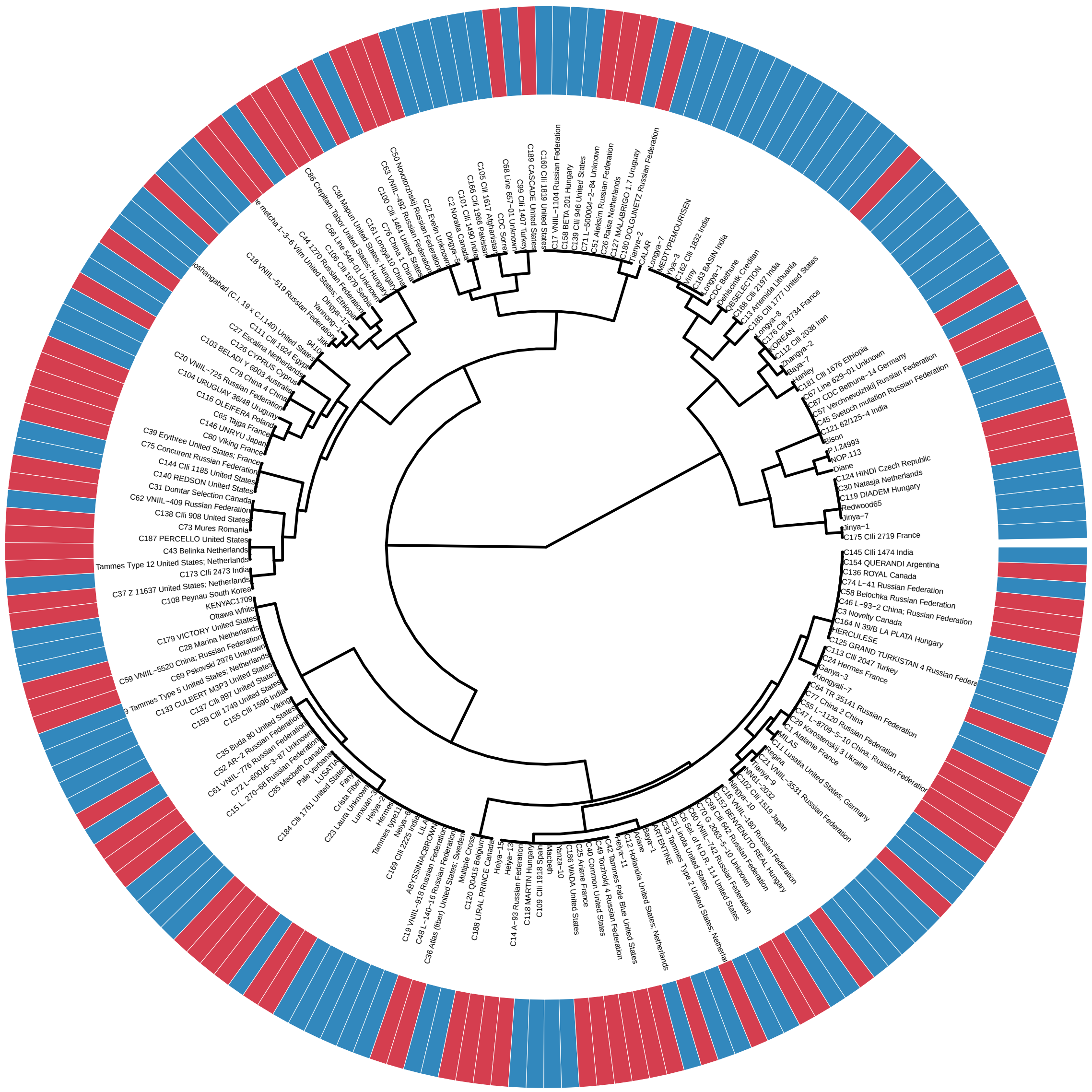

Supplementary Figure S4q. Dendrogram of linseed and fiber flax varieties based on polymorphisms of *ABCB47* gene.

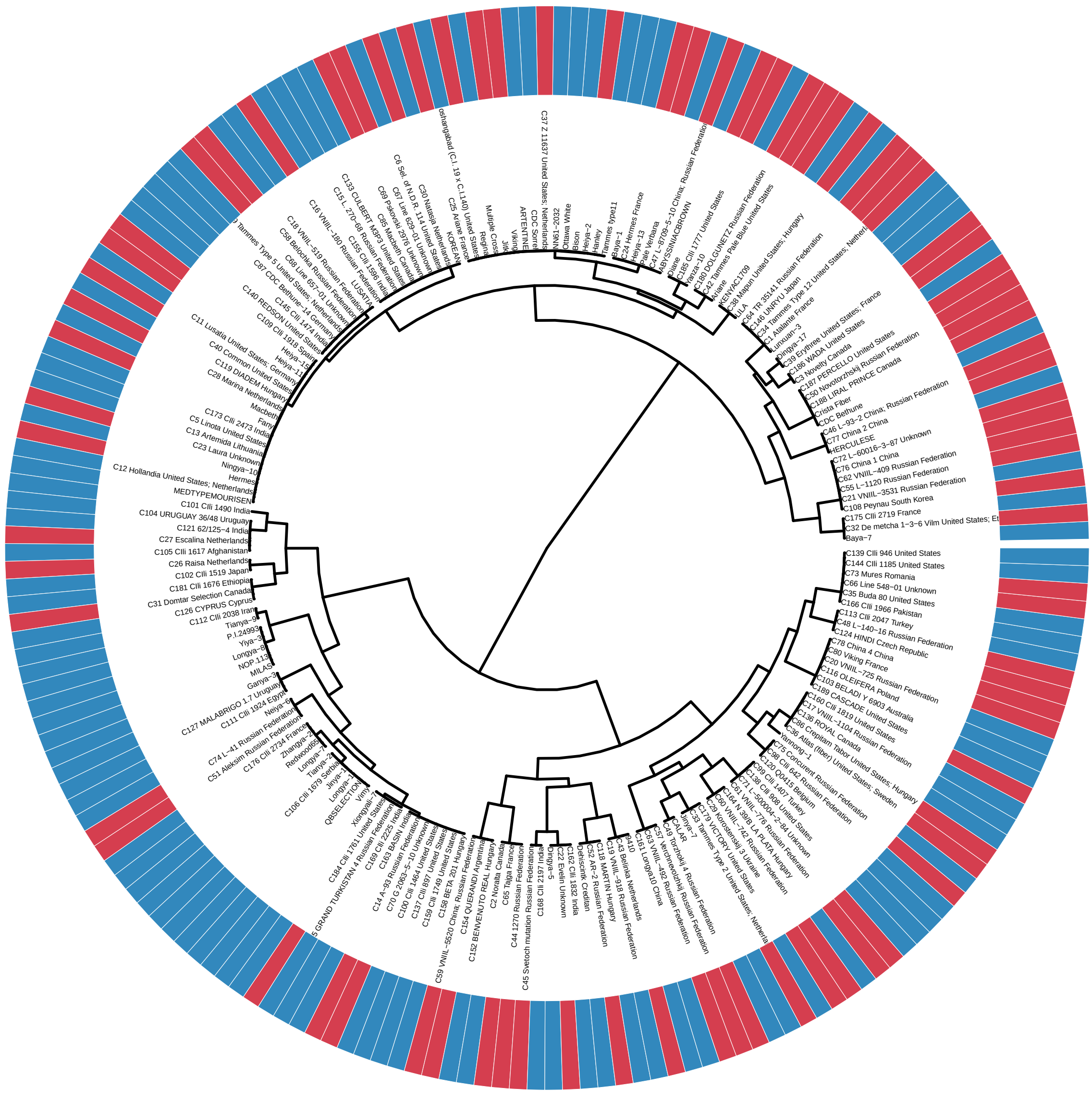

Supplementary Figure S4r. Dendrogram of linseed and fiber flax varieties based on polymorphisms of *ABCG71* gene.

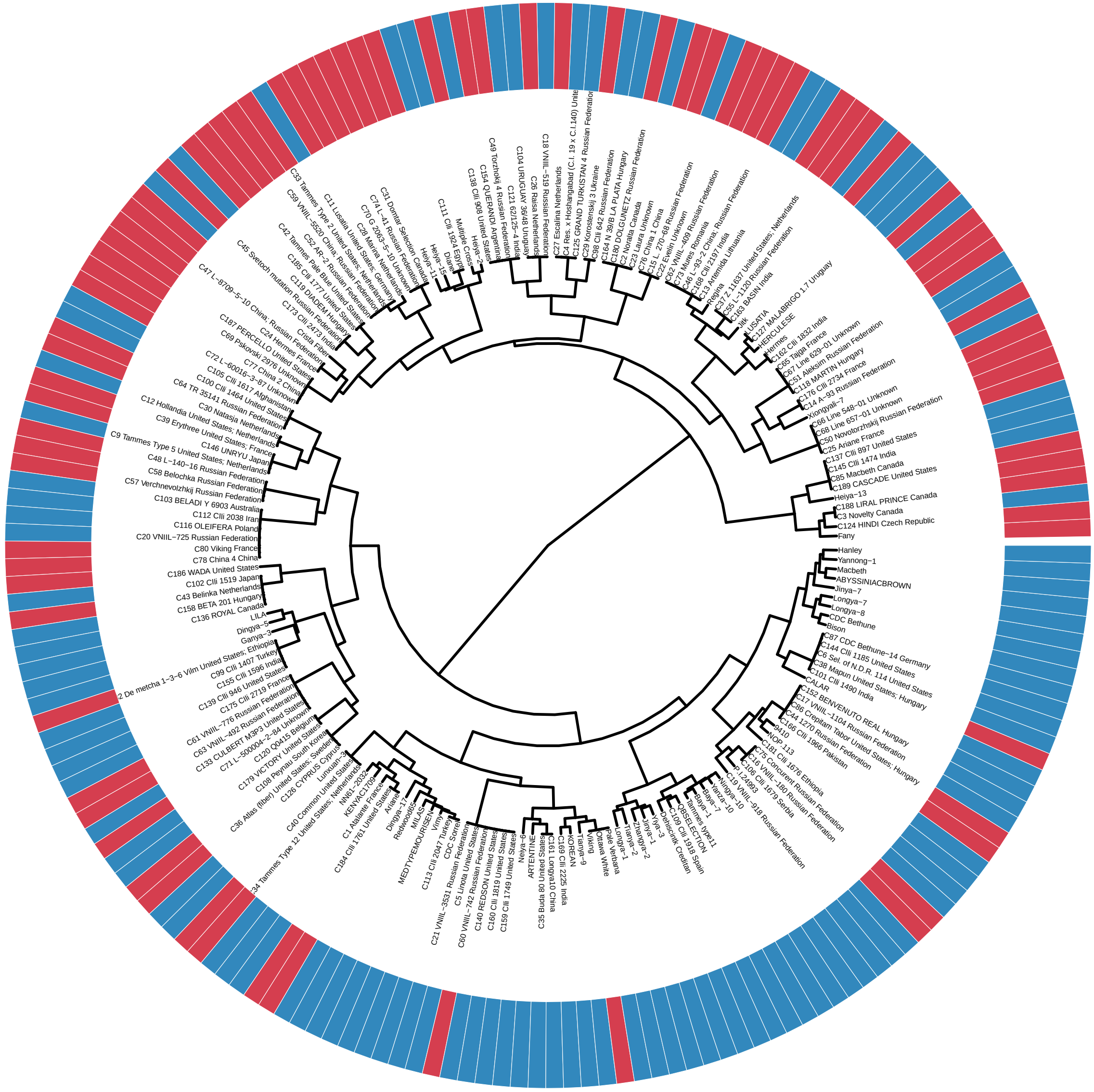

Supplementary Figure S4s. Dendrogram of linseed and fiber flax varieties based on polymorphisms of *ABCG80* gene.

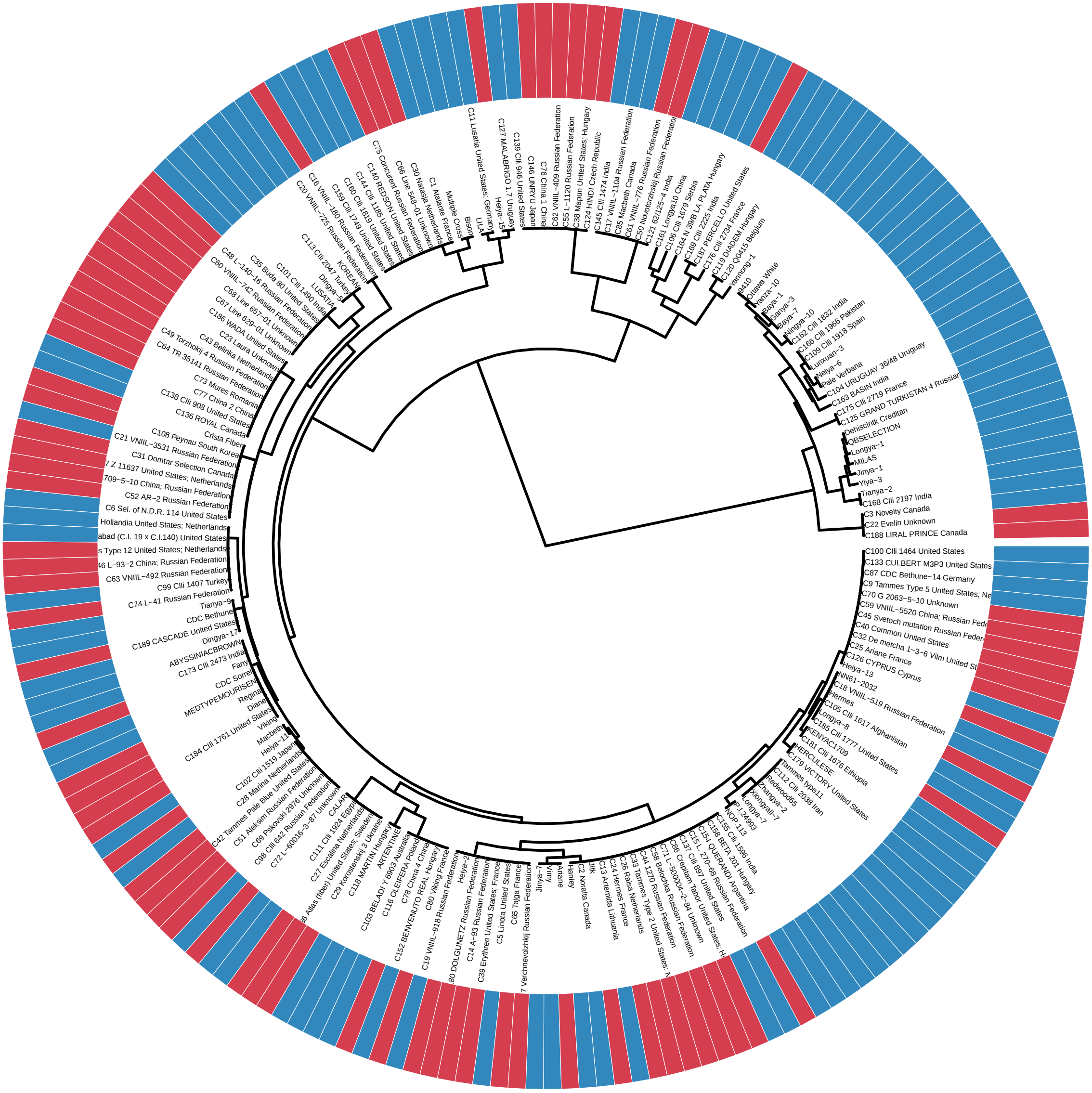

Supplement: Supplementary file 1 [file plants-10-02616-s001.zip › Supplementary Figure S4.pdf]
